# A Melanoma Brain Metastasis CTC Signature and CTC:B-cell Clusters Associate with Secondary Liver Metastasis: A Melanoma Brain–Liver Metastasis Axis

**DOI:** 10.1158/2767-9764.CRC-24-0498

**Published:** 2025-02-12

**Authors:** Tetiana Y. Bowley, Mireya C. Ortiz, Irina V. Lagutina, Mara P. Steinkamp, Bridget N. Fahy, Bernard Tawfik, Moises Harari-Turquie, Dario Marchetti

**Affiliations:** 1 Division of Molecular Medicine, Department of Internal Medicine, University of New Mexico Health Sciences Center, Albuquerque, New Mexico.; 2 Animal Models Shared Resource, The University of New Mexico Comprehensive Cancer Center, Albuquerque, New Mexico.; 3 Department of Pathology, University of New Mexico Health Sciences Center, Albuquerque, New Mexico.; 4 Division of Surgical Oncology and Palliative Medicine, University of New Mexico Comprehensive Cancer Center, Albuquerque, New Mexico.; 5 Division of Hematology and Oncology, Department of Internal Medicine, University of New Mexico Comprehensive Cancer Center, Albuquerque, New Mexico.

## Abstract

Melanoma brain metastasis is linked to dismal prognosis and low overall survival and is detected in up to 80% of patients at autopsy. Circulating tumor cells (CTC) are the smallest functional units of cancer and precursors of fatal metastasis. We previously used an unbiased multilevel approach to discover a unique ribosomal protein large/small subunit (RPL/RPS) CTC gene signature associated with melanoma brain metastasis. In this study, we hypothesized that CTC-driven melanoma brain metastasis secondary metastasis (“metastasis of metastasis” per clinical scenarios) has targeted organ specificity for the liver. We injected parallel cohorts of immunodeficient and newly developed humanized NBSGW (huNBSGW) mice with cells from CTC-derived melanoma brain metastasis to identify secondary metastatic patterns. We found the presence of a melanoma brain–liver metastasis axis in huNBSGW mice. Furthermore, RNA sequencing analysis of tissues showed a significant upregulation of the RPL/RPS CTC gene signature linked to metastatic spread to the liver. Additional RNA sequencing of CTCs from huNBSGW blood revealed extensive CTC clustering with human B cells in these mice. CTC:B-cell clusters were also upregulated in the blood of patients with primary melanoma and maintained either in CTC-driven melanoma brain metastasis or melanoma brain metastasis CTC–derived cells promoting liver metastasis. CTC-generated tumor tissues were interrogated at single-cell gene and protein expression levels (10x Genomics Xenium and HALO spatial biology platforms, respectively). Collectively, our findings suggest that heterotypic CTC:B-cell interactions can be critical at multiple stages of metastasis.

**Significance:**

This study provides important insights into the relevance of prometastatic CTC:B-cell clusters in melanoma progression, extends the importance of the CTC RPL/RPS gene signature beyond primary metastasis/melanoma brain metastasis driving targeted organ specificity for liver metastasis (“metastasis of metastasis”), and identifies new targets for clinical melanoma metastasis therapies.

## Introduction

Melanoma is the most aggressive skin cancer which associates with high metastatic burden, especially to the brain (1–4). Melanoma brain metastasis is detectable in 60% of advanced melanoma cases and in up to 80% at the time of autopsy (5–7). Melanoma brain metastasis represents the third most common origin of brain metastasis, surpassed only by lung and breast cancers (8, 9), and is increasing in frequency. Patients with melanoma brain metastasis have dismal survival rates and most will die of their disease in 4 to 6 months (10, 11). The treatment of early-stage melanoma tumors includes surgical resection and radiation followed by adjuvant therapies, whereas metastatic melanoma is treated with systemic therapies. Although immunotherapies and targeted therapies have improved the prognosis of these patients, overall patient survival remains poor, and novel biomarkers/therapeutic approaches are desperately needed (10, 12, 13).

Cancer cells with unique properties intravasate into the bloodstream [circulating tumor cells (CTC)] and initiate tumor formation at distant organs via a complex set of events (14–16). CTCs are the smallest functional units of cancer and “seeds” of fatal metastasis. CTCs encounter extensive microenvironmental, shear, and oxidative stresses in blood, and 99% die early prior to successful metastatic onset (17, 18). However, subsets of CTCs can acquire a quiescent state and reside in the bone marrow for years before their activation (18, 19). CTCs can shed from the original primary lesion, thus promoting metastasis via their dissemination in blood, arrest, adhesion, and extravasation at distant organs, such as the liver, lung, skin, and brain. Of note, CTCs can also intravasate from established metastases to further promote metastatic dissemination (“metastasis of metastasis”: the metastatic cascade; refs. 14, 20–22). Clinical metastasis therefore occurs at multiple sites sequentially and/or concurrently and results in poor clinical outcomes and high mortality rates (23). Importantly, the number of CTCs in patients’ blood directly relates to cancer severity, metastasis, and overall patient survival (15, 24). Furthermore, although CTC clusters (two or more cells) comprise only 2% to 5% of all CTCs, their presence in blood indicates dramatically increased metastatic capabilities among cancer types, e.g., 23 to 50 times higher than single CTCs which translates in poor patient survival (25–27).

CTC gene profiles are distinct from primary versus metastatic tumors and represent the gain of novel mutations or properties, allowing for increased metastatic competence (19, 28). Growing melanoma brain metastasis onset and burden enable enhanced CTC shedding into patients’ bloodstream. Previously, we used an unbiased, multipronged approach which resulted in the discovery of a unique 21-member 60S large ribosomal unit (RPL)/40S small ribosomal unit (RPS) CTC gene signature associated with melanoma brain metastasis, and its progression in the brain was investigated spatially and temporally (1, 29, 30). Furthermore, we found four RPL/RPS members of the melanoma CTC signature (RPL23, RPL35A, RPL6, and RPS18) of 21 RPL/RPS genes to be keenly involved in metastatic progression. Drug inhibitory studies demonstrated that the dual targeting of cell translation/cell proliferation inversely affected CTC glycolytic metabolism in real time; however, it was critical to suppress CTC metastasis in immunodeficient mice (31). CTCs utilize aberrant ribogenesis to preferentially translate oncogenic RNA transcripts to initiate metastatic dissemination and progression, and enhanced ribosomal RPL15 synthesis was reported to be directly linked to breast cancer metastasis (32, 33). Moreover, the trend of enhanced tumor-specific total mRNA synthesis has been observed in 6,580 patients across 15 different cancer types, which correlates with poor clinical prognosis (34).

It is noteworthy that multiple studies have identified heterotypic clustering of CTCs with normal cells, including neutrophils, polymorphonuclear myeloid-derived suppressor cells (PMN-MDSC), CD3 cells, CD4 cells, and platelets in metastatic patients (25–27, 35). For example, the presence of as little as one CTC–neutrophil cluster in patient’s blood was linked to lower survival rates and worse clinical outcomes when compared with that in patients with no heterotypic CTC clusters (35).

Here, we provide for the first time evidence that CTC-driven primary melanoma brain metastasis has a liver-targeted organ specificity to generate extensive secondary metastasis to the liver, thus discovering the presence of a CTC brain–liver metastasis axis. Second, we report the presence of CTC:B-cell clusters and detection of high number of these clusters in blood of patients with primary melanoma with no clinical evidence of metastasis. Conversely, CTC evaluation of blood of patients with metastatic melanoma revealed a 15/20-fold decrease in CTC:B-cell clusters. These findings implicate their relevance early in the metastatic cascade and substantiate notions that melanoma B-cell phenotypes interacting with CTCs can be prometastatic and predict disease severity and clinical outcomes (36, 37). Third, by using a unique humanized NBSGW (huNBSGW) mouse model which possesses high peripheral blood B-cell numbers (38, 39), we provide evidence for CTC:B-cell implications in melanoma progression, e.g., from primary melanoma brain metastasis to secondary metastatic disease, closely reflecting the clinical scenario (“metastasis of metastasis”). To this end, first-generation CTC xenografts (CDX) were generated by intracardiac injection of melanoma brain metastasis–competent CTCs, whereas second-generation CDX mice were developed by engrafting blood or melanoma brain metastasis cells from the first-generation, mice revealing the presence of CTC:B-cell clusters in both generations. Lastly, we report that these pivotal interactions are present either at the level of blood or brain and liver tissues by using the cutting-edge single-cell 10x Genomics Xenium (gene expression) and Indica HALO (protein expression) spatial biology platforms. The extensive liver metastasis observed in the second-generation NBSGW (NOD.Cg-KitW-41J Tyr + Prkdcscid Il2rgtm1Wjl/ThomJ) mice injected with melanoma brain metastasis–derived cells had highly elevated expression of the RPL/RPS gene CTC signature of melanoma brain metastasis, fostering the relevance of this signature in the brain–liver axis of melanoma. Altogether, these findings provide a conduit for therapeutic targeting of CTC:B-cell clusters which could inhibit metastasis and thus render more patients curable.

## Materials and Methods

### Patient blood collection

Blood samples from patients with either primary melanoma or metastatic melanoma were gathered according to the protocols approved by the Institutional Review Board at the University of New Mexico (UNM) Health Sciences Center in Albuquerque, New Mexico. Primary melanoma was defined as tumor confined to the original site of disease that was resected with curative intent. Blood was taken prior to definitive surgical resection of the primary tumor and nodal staging. Conversely, metastatic melanoma refers to blood taken from patients with tumor spreading to the brain, liver, lung, and other skin locations outside the original site. Clinical information about each patient is provided. Patients signed written informed consent before the scheduled sample collection. Using the aseptic technique, patients’ peripheral blood (15–25 mL) was drawn from the middle of the vein puncture into an EDTA tube as part of their medical appointment. Samples were transported to the laboratory for the isolation and interrogation of CTCs. These samples were either processed immediately or preserved in CellSave (Menarini Silicon Biosystems, Inc.) or Streck Cell-Free DNA BCT tubes (Streck).

### Isolation of single CTCs and CTC clusters

Blood from mice (100–300 μL) was drawn retro-orbitally using an EDTA-coated glass pipette and transferred into Greiner MiniCollect collection tubes (Greiner Bio-One, Cat. #K3E K3EDTA). Blood was then loaded onto the FDA-approved CTC Parsortix microfluidic chip (pore size 6.5 μm) within an hour or two of necropsy. The Parsortix PR1 Platform (Angle Europe Ltd.) with a 6.5-M cartridge (Angle, PLC) was used to capture single CTCs and CTC clusters through filtration and microfluidic methods. CTCs were identified as cells stained for human MelA (labeled with Alexa Fluor 594, Santa Cruz Biotechnology, Cat. #sc-20032), human CD45 (FITC-tagged, BioLegend, Cat. #103108), and 4′,6-diamidino-2-phenylindole (DAPI; Thermo Fisher Scientic, Cat. #D3571). CD19 antibody [mAb (H1B19), Thermo Fisher Scientific, eBioscience, Cat. #14-0199-82] was used to stain B cells. CTCs were observed and counted using a Zeiss LSM800 microscope (10× and 40× magnification) and analyzed with ZEN Blue 2.1 software (Carl Zeiss Microscopy).

### Cell culture

Highly metastatic brain melanoma CTC–derived clonal cells (70W-SM3; generated in Dr. Marchetti’s laboratory) were grown as previously described (1). The luciferase-tagged CTC clone cells (70W-SM3-Luc2) were prepared as previously described (40). Briefly, CTC-derived clonal cells were cultured with DMEM/F12 (Thermo Fisher Scientific, Cat. #11320081) supplemented with 10% FBS (Gibco, Cat. #A4766801) and 1× GlutaMAX (Gibco, Cat. #35050061). The cells were maintained in a humidified chamber at 37°C with 5% CO_2_. The cells were passaged using Accutase (STEMCELL Technologies, Cat. #07922) or 0.05% trypsin-EDTA (Gibco, Cat. #25300054) before reaching their maximum confluency.

### HuNBSGW mouse model

HuNBSGW mice were engrafted by the UNM Comprehensive Cancer Center Animal Models Shared Resource. Frozen human CD34^+^ cord blood cells from mixed donors were purchased from HumanCells Bio (Cat. #CBCD34-mix-C1M). One million cells were thawed according to the manufacturer’s recommendations. The cells were then washed twice with Stemline II Hematopoietic Stem Cell Expansion Medium (S0192, Sigma-Aldrich). The donor cell pellet was resuspended in 1 mL of StemSpan SFEM II medium (09605, STEMCELL Technologies Inc.) supplemented with 0.1 μg/mL Human Recombinant SCF (78062.1, STEMCELL Technologies Inc.) prior to cell counting. The cells were then seeded in a 24-well plate and allowed to recover overnight (12 hours) in a cell culture incubator (at 37°C with 5% CO_2_ ). Afterward, CD34^+^ cord blood cells were resuspended in PBS and injected into the livers of 1- to 3-day-old NBSGW mice as described (41). The humanization rate (% human CD45^+^ cells in peripheral blood) was assessed at 12 weeks after injection by flow cytometry with an antibody panel including human-specific (clone HI30, BD Biosciences) and mouse-specific (clone 30-F11, BD Biosciences) anti-CD45 antibodies as described previously (38).

### CDXs

Mouse studies were approved by the UNM Institutional Animal Care and Use Committee. CDXs were generated using 6- to 12-week-old NBSGW mice. A total of 24 huNBSGW and 24 NBSGW mice were injected retro-orbitally with 50 μL (4 mg/mL) of low–molecular weight heparin (NDC, Cat. #63323-533-01) prior to intracardiac injection to avoid thromboembolism. Mice were anesthetized with isoflurane (2.5%, 1 L/minutes O_2_ flow), and 5.0 × 10^5^ CTC-derived clonal cells (passage 18) were injected into the left ventricle of the heart using a sterile 0.5-mL U-100 insulin syringe with a 29G x 1/2 inch needle (Becton Dickinson, Cat# 58324702). Blood backflow confirmed successful intracardiac needle placement. Longitudinal bioluminescent imaging of tumor burden using the IVIS Spectrum *in vivo* imaging system (PerkinElmer) was performed weekly to monitor tumor development in mice. After 6 weeks, five melanoma brain metastasis male and five melanoma brain metastasis female mice from both cohorts (humanized and nonhumanized) were euthanized and necropsied. Samples from huNBSGW with melanoma brain metastasis were reinjected into a second generation of huNBSGW hosts, whereas samples from non-huNBSGW with melanoma brain metastasis were reinjected into non-huNBSGW hosts. Blood was collected and reinjected retro-orbitally (350 μL) into second-generation mice (“blood-derived” mice) in both humanized and nonhumanized NBSGW cohorts. Brain tumors were isolated based on their black-to-brown pigment, dissociated using Accumax (STEMCELL Technologies, Cat. #07921), and engrafted as a single-cell suspension of 5.0 × 10^5^ cells in PBS by intracardiac injection into the second-generation of mice (“brain-derived” mice). A schematic recapitulating the experimental plan is shown in Supplementary Fig. S3. All mice were monitored daily for signs of lethargy and distress, such as sudden weight loss, unkempt fur, difficulty with breathing, seizures, and neurologic problems. At a humane endpoint, mouse blood was collected into an EDTA-containing MiniCollect tube (Greiner Bio-One, Cat. #K3E K3EDTA). CTCs were isolated using the FDA-approved CTC Parsortix platform, and RNA was extracted for further RNA sequencing (RNA-seq). The brain, liver, lungs, and spleen were fixed (10% formalin) for Xenium (10x Genomics) and HALO (Indica Lab) analyses.

### RNA-seq

Following Parsortix CTC capture (Angle, LLC, Plymouth Meeting), RNA was isolated from enriched and flow-through fractions (10–20 × 10^3^ cells). The enriched fraction was comprised of human CTCs, whereas the flow-through fraction contained immune cells. The mRNA was extracted using RNeasy Micro Kit (Qiagen, Cat. #74004) following the manufacture’s protocol. The RNA was analyzed, cDNA was synthesized, and library preparation was performed using the microarray platform SMARTer Universal Low Input RNA Kit for RNA-seq (Clontech, Cat. #634946). Fragmented RNA underwent processing using Ion Plus Fragment Library Kit (Thermo Fisher Scientific, Cat. #4471252). The samples were sent for sequencing on the Ion Proton S5/Xl platform (Thermo Fisher Scientific) at the Analytical and Translational Genomics Shared Resource Core located at the UNM Comprehensive Cancer Center.

### Bioinformatic and biostatistical analyses

Tmap (v5.10.11) was used to align RNA-seq analysis data to a BED file format with nonoverlapping exon regions. Exons were quantified using HTSeq (v0.11.1; ref. 42). Counts across exons were averaged to generate gene-level counts. EdgeR was used to normalize the size of the library and to perform differential analysis (43, 44). Heatmap3 was utilized to generate heatmaps and cluster analyses. Enrichment of pathways was conducted through the Reactome database, as previously described (45).

### 10x Genomics Xenium analysis

Tissue sections were processed using the manufacturer’s guidelines for Xenium (10x Genomics; www.10xgenomics.com). Both the standard predesigned human multitissue/cancer panels (10× Genomics, product number 1000626), as well as a custom-designed panel [30 customized genes, including all 21 members of the RPL/RPS CTC signature (B)], were used to generate the 10× Xenium results. Decoding of optical transcript signals and cell segmentation were performed using the on-board software (v2.0.0.10), resulting in transcript assignment to cells. The resulting count and image files were further analyzed with Seurat (v. 5.0.1) to visualize clustering and custom transcript location (46, 47).

### Indica HALO analysis

Following necropsy, liver and brain tissues from first- and second-generation NSBGW and huNBSGW mice were fixed immediately in 10% formalin. Fixed tissues were paraffin-embedded and sectioned. To detect infiltrating immune cells, IHC was performed on the Ventana Discovery Ultra Platform using immunoperoxidase labeling by the UNM Human Tissue Repository. The slides were labeled with hematoxylin as a nuclear counter stain for cell identification, and Roche DISCOVERY Purple chromogen staining was performed to identify MelanA. All tissue slides were scanned using the Leica Aperio AT2 digital scanner at 20× magnification. Quantitative analysis of MelanA-positive cells and immune cell infiltration was performed using the HALO analysis and annotation software package (Indica Labs). MelA was consistently expressed in melanoma brain metastasis/liver xenograft tissues (Fig. 8; ref. 19). Notably, we consistently detected high melanin content in melanoma brain metastasis and other metastatic lesions, which suggests a direct correlation between MelA expression in patients and aggressive disease. Furthermore, the HALO system parameter for positivity intensity was set to the optical density threshold of 0.02. The system’s Figure Maker feature was used to generate the magnification series of images with and without HALO markup.

### Data availability

The data generated in this study are publicly available in Gene Expression Omnibus at GSE280739 and GSE280741 per NIH guidelines and schedule.

## Results

### Establishment of the humanized melanoma brain metastasis CDX model

Interrogating the importance of the immune system in melanoma progression and metastatic onset enabled us to generate the first humanized melanoma brain metastasis CDX model. Previously, we reported the development of the first immunodeficient CDX melanoma model (1). We used nonirradiated huNBSGW animals to generate a more clinically relevant model (38). HuNBSGW mice (12 males and 12 females) were injected with CTC-derived Luc-tagged clonal cells, as previously reported (1, 31). The CTC clonal cell line was tagged with the Luc2 protein to monitor metastatic spread and change in tumor burden over time by bioluminescence imaging and to optimize collection time points for IVIS (*In Vivo* Imaging System) imaging. The CTC-derived clone is highly aggressive, with melanoma brain metastasis detected as early as 24 hours following intracardiac injection (1). It has been reported that the expression of foreign proteins such as luciferase may affect immune response. However, this effect is not only highly dependent on the cell line (48), but also on the mouse model used. Parallel experiments with intracardiac injections of the Luc2 CTC-derived clonal line (70W-SM3 cells) in immunocompromised [NOD/SCID gamma (NSG) or NBSGW] and huNBSGW mice showed that the time from injection to fully developed metastatic melanoma, which required humane euthanasia, was the same (no statistical difference), suggesting that immunogenicity of Luc2 under our experimental conditions was negligible. Successful engraftment of immune system cells was confirmed by flow cytometry and by the presence of tumor-infiltrating lymphocytes and tumor-associated macrophages in brain/liver metastases by tissue immunostaining for CD3, CD4, CD8, CD68, human mitochondrial marker, and CD19 immune biomarkers (Supplementary Fig. S1A; ref. 38). The percentage of infiltrating human immune cells (number of positive-stained cells/total number of cells in the section) was computed using HALO software (*N* = 3; Supplementary Fig. S1B). Tissues from mice with similar humanization levels were used to perform HALO analyses. Due to difficulty in humanizing the male mice, several huNBSGW mice had lower than 20% humanization rates; however, even mice with lower humanization rates had infiltrating immune cells in metastases and showed difference in metastatic patterns compared with nonhumanized mice. No clinical symptoms of GVHD were observed in any of the huNBSGW animals. Importantly, the level of humanization of first- and second-generation mice was adequate to perform studies described (Supplementary Table S1).

**Figure 1:**
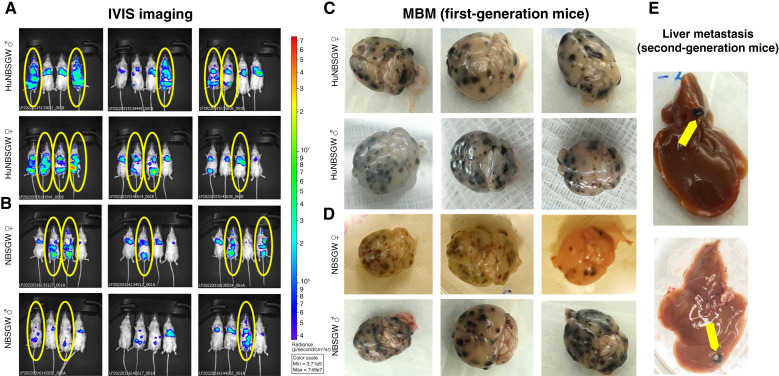
Metastatic patterns of first-generation CDX mice by IVIS imaging. Gender-specific huNBSGW (**A**) and immunodeficient NBSGW (**B**) mice were engrafted with CTC-derived clonal cells by intracardiac injection (70W-SM3-Luc2; 5.0 × 10E5/animal). IVIS imaging was performed every week to evaluate metastatic onset. The last IVIS time point (5 weeks) is shown. Mice with MBM are circled. Following necropsy, pathologic evaluation revealed multifocal MBM in huNBSGW (**C**) and NBSGW (**D**) groups. MBM (*N* = 5 per group) were selected for tumor cell dissociation, isolation, and reinjection to obtain the second-generation CDXs. **E,** Representative MBM-generated liver metastasis in second-generation huNBSGW CDX mice. See “Materials and Methods” for experimental details. MBM, melanoma brain metastasis.

A cohort of 24 immunodeficient NBSGW mice was engrafted with CTC-derived clonal cells in parallel. Metastasis occurrence and progression were monitored by weekly IVIS imaging. A percentage of humanized and immunodeficient mice presented with melanoma brain metastasis (Fig. 1A and B). The quantification of melanoma brain metastasis tumor burden (total flux) by IVIS imaging confirmed time-dependent melanoma brain metastasis progression in both models (Supplementary Fig. S2). Notably, animal necropsies had extensive melanoma brain metastasis in huNBSGW and NBSGW mice (Fig. 1C and D). Additionally, melanoma cells disseminated to the liver (Fig. 1E), lungs, intestine, bone marrow, and spleen, recapitulating target organ metastatic specificity of clinical melanoma (49). This set of animals was designated “first-generation” mice.

At the end of the study, melanoma brain metastasis tumors from 10 huNBSGW mice were dissociated and engrafted by intracardiac injection as a single-cell suspension into “second-generation” huNBSGW mice to interrogate the site preference of melanoma brain metastasis cancer cells. Likewise, melanoma brain metastasis from non-huNBSGW mice was engrafted into second-generation non-huNBSGW mice. In parallel, another cohort of mice was injected with blood from the first-generation mice to compare metastatic target organs of blood-residing CTCs with those of melanoma brain metastasis tumor cells. Humanization in the second-generation mice was confirmed prior to the engraftment of blood/melanoma brain metastasis cells (Supplementary Table S2). A flowchart depicting the detailed outline of experimental procedures is presented in Supplementary Fig. S3.

### Melanoma brain metastasis–competent CTCs promote extensive secondary liver metastasis

**Figure 2:**
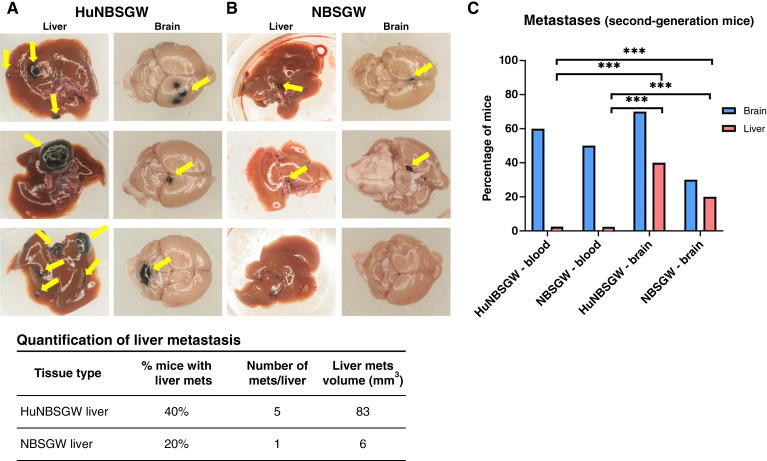
The melanoma brain–liver metastasis axis. **A** and **B,** MBM-generated liver metastasis and MBM of second-generation mice. MBM/liver metastasis (yellow arrows) from second-generation huNBSGW (**A**) and NBSGW (**B**) mice engrafted via intracardiac injection with first-generation CDX MBM cells (5.0 × 10E5 cells/animal). Quantification of liver metastasis shows higher %, number, and size of liver metastasis in huNBSGW mice (bottom). **C,** Statistical significance (***, *P* value < 0.001) for the presence of brain (MBM) and liver metastases, showing distinct onset in the second-generation CDXs injected with blood- vs. MBM-dissociated cells from first-generation CDXs. See “Materials and Methods” for experimental details. MBM, melanoma brain metastasis.

IVIS quantification of the melanoma brain metastasis signal was performed (Supplementary Fig. S4). The second-generation mice were necropsied 8 weeks after injection of brain cells or blood. Importantly, major differences were detected in metastatic patterns in the presence or absence of human immune cells in these mice. First, second-generation huNBSGW mice engrafted with melanoma brain metastasis developed extensive liver metastases (Fig. 2A) which were significantly larger in size and numbers than second-generation NBSGW (Fig. 2B) or first-generation huNBSGW liver tumors. Second, blood-injected animals, regardless of immune competency, did not develop any liver metastases (Fig. 2C). Interestingly, blood-injected CDXs developed melanoma brain metastasis earlier than brain-injected (melanoma brain metastasis–derived cells) CDXs. Third, there were differences in melanoma brain metastasis occurrence in the presence of human immune cells. A higher number of huNBSGW mice developed melanoma brain metastasis than immunodeficient animals [40% vs. 20% melanoma brain metastasis mice; Fig. 2A and B (bottom)]. The comparison of all metastatic sites between the two groups is presented in Supplementary Table S3.

**Figure 3:**
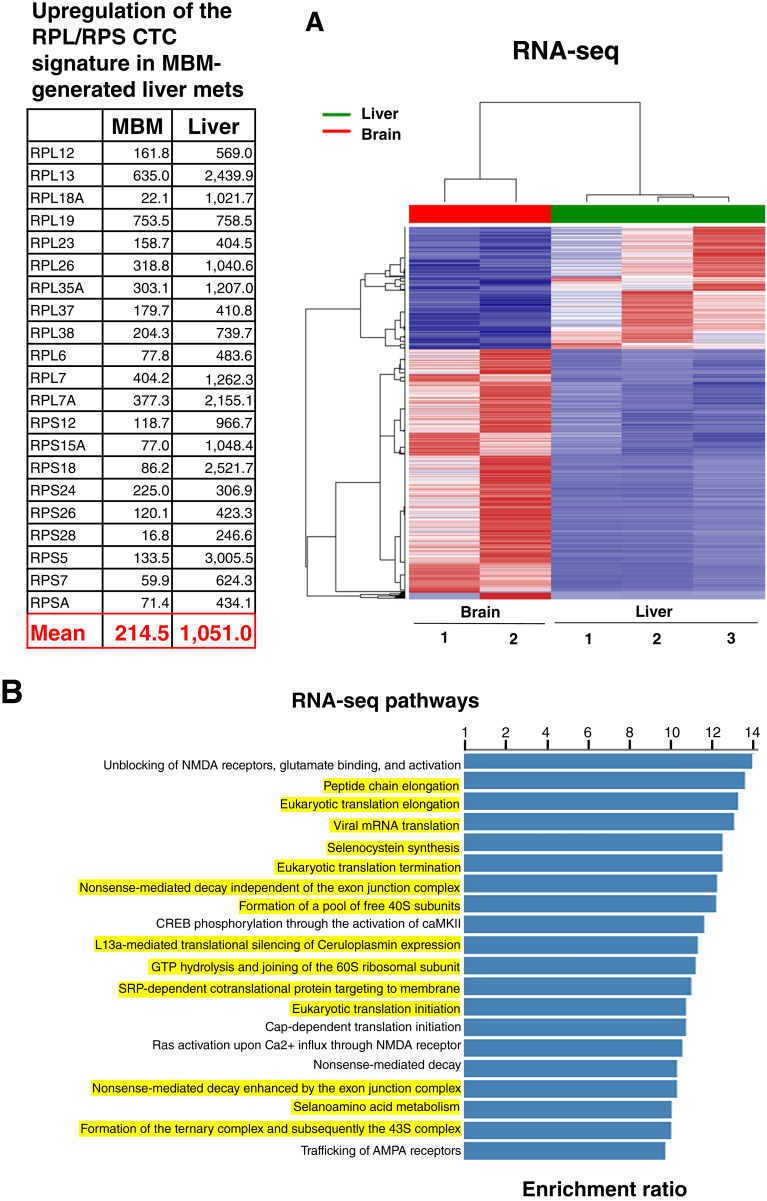
Commonality and upregulation of the MBM RPL/RPS CTC gene signature in the second-generation huNBSGW CDXs. **A,** RNA-seq gene expression profiling showing distinct clustering patterns between cells of liver (green) and brain (red) metastasis (right). Upregulation of the CTC RPL/RPS gene signature in MBM-generated liver metastasis vs. MBM is shown (left) as calculated in counts per million. Mean values of the 21 RPL/RPS members of the CTC signature are indicated (red). **B,** Statistically significant RNA-seq pathways generated by Reactome database analysis. Pathways highlighted in yellow are the same of RPL/RPS CTC gene expression pathways (1). AMPA stands for α-amino-3-hydroxy-5-methyl-4-isoxazolepropionic acid. See “Materials and Methods” for experimental details. MBM, melanoma brain metastasis.

Previously, we used an unbiased and multipronged approach to identify a specific 21-member RPL/RPS melanoma CTC signature associated with melanoma brain metastasis (1). To investigate it further, we performed RNA-seq on brain- and liver-dissociated cells isolated from second-generation huNBSGW mice injected with brain tumor cells. Transcriptional profiling of brain- and liver-derived tumor cells showed distinct clustering patterns in brain tumor versus liver tumor cells [Fig. 3A (right)]. Intriguingly, huNBSGW liver cells had a five-fold increase in the RPL/RPS gene expression compared with brain cells from the same mice [Fig. 3A (left)]. This observation suggests that brain-derived melanoma cells target the liver in which they establish a tumor-promoting niche and develop extensive liver metastasis. Thus, there may be a brain–liver axis in melanoma. The Reactome pathway software analyzed differential gene expression between samples and produced a list of statistically significant pathways (Fig. 3B). Importantly, the majority of differential pathways were CTC RPL/RPS gene pathways (highlighted in yellow; ref. 1). These translational pathways play a critical role in cancer progression (50).

### B-cell clustering with melanoma brain metastasis–competent CTCs

**Figure 4:**
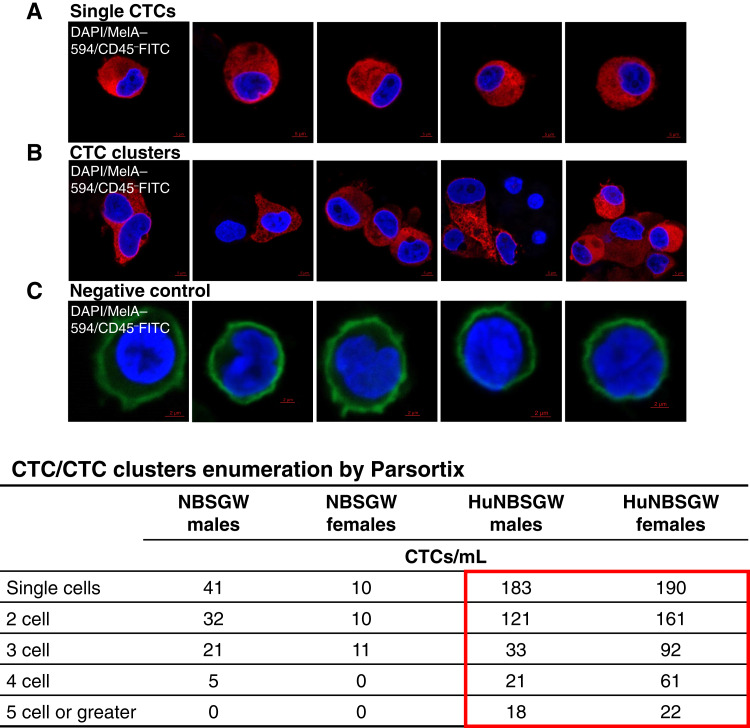
Capture and interrogation of CTCs from huNBSGW and NBSGW CDXs by the FDA-approved CTC Parsortix platform. Blood of the second-generation CDXs was collected at the end of the study and immunostained for visualization of single CTCs (MelA^+^/DAPI^+^/CD45^−^ cells; **A**) and CTC clusters (**B**). **C,** Cells from healthy donor blood (MelA^–^/DAPI^+^/CD45^+^) were stained in parallel as control. Bottom, model-specific, gender-specific number of single CTCs and CTC clusters (two-cell or greater) quantified by high-definition confocal microscopy in second-generation CDXs. A significant increase of CTCs/CTC clusters was detected in huNBSGW CDXs, regardless of gender (red box). See “Materials and Methods” for experimental details.

To quantify CTC numbers and identify CTC traveling partners, we captured and analyzed CTCs from immunodeficient and huNBSGW groups using the CTC Parsortix microfluidic platform. In each group (Supplementary Fig. S3 flowchart), an equal volume of pooled blood was analyzed via Parsortix to capture and isolate human CTCs based on their size and deformability. Individual CTCs and CTC clusters were immunostained for human CD45-FITC, human MelanA Alexa 594, and DAPI nuclear marker. MelA^+^/DAPI^+^/CD45^−^ cells were defined as melanoma CTCs which were imaged via confocal microscopy and enumerated (Fig. 4A and B). Healthy donor blood was used as a negative control to confirm the presence of CD45^+^ cells and the absence of melanoma CTCs (Fig. 4C). A considerable increase (up to a ∼20-fold difference between huNBSGW and NBSGW) of single CTCs and CTC clusters was detected in the blood of huNBSGW mice [Fig. 4C (bottom)]. The presence of CTC clusters indicates strong metastatic competence and cancer severity (26, 27). Importantly, both homotypic and heterotypic CTC clusters were detected in the blood of huNBSGW mice (Fig. 4B). CD45^+^ staining was used to identify CTC clustering with white blood cells; interestingly, CTC traveling partners were CD45^−^ in CTC clusters.

**Figure 5:**
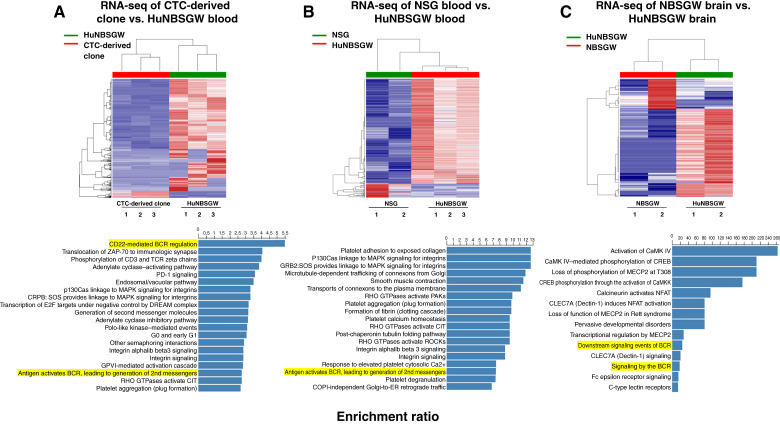
RNA-seq analyses of blood and brain from second-generation huNBSGW and NBSGW mice. **A** and **B,** CTCs from blood of second-generation huNBSGW CDXs (*N* = 3) were harvested via Parsortix, followed by RNA-seq interrogation. Distinct unsupervised hierarchical clustering of gene expression between huNBSGW CTCs and uninjected CTC-derived clonal cells (**A**) or blood-derived CTCs from immunodeficient NSG mice (**B**). **C,** RNA-seq analyses of cells isolated from MBM cells of second-generation huNBSGW/NBSGW CDXs with heat-maps showing differential clustering of MBM-derived cells in the presence or absence of human immune cells. Bottom, Top molecular pathways resulting from comparisons in huNBSGW vs. NBSGW CDXs (Reactome pathway database). All RNA-seq analyses showed the presence of B cell–related pathways in huNBSGW CDXs (highlighted in yellow). See “Materials and Methods” for experimental details. MBM, melanoma brain metastasis.

For further interrogation of heterotypic CTC clusters, blood from three melanoma brain metastasis huNBSGW mice was collected via retro-orbital draw. Parsortix was used to capture and harvest single CTCs and CTC clusters for single-cell RNA-seq analysis. Unsupervised hierarchical clustering between CTCs and uninjected CTC clonal cells showed obvious distinctions in clustering patterns between samples [Fig. 5A (top)]. Analysis of differential gene expression via the Reactome database generated a list of molecular pathways, including immune pathways [Fig. 5A (bottom)]. Importantly, the most statistically significant pathway was a B cell–related pathway “CD22-mediated B-cell receptor (BCR) regulation” that was upregulated in CTC clusters from huNBSGW blood. A list of the 20 most statistically significant pathways included another B cell–related pathway, additive to previously reported interactions of CTCs with PMN-MDSCs, platelets, neutrophils, CD3, and CD4 cells in CTC clusters (26, 27, 35). As uninjected CTC-derived clonal cells did not contain any immune cells, the presence of B-cell pathways in this analysis showed B-cell clustering specificity with melanoma brain metastasis–competent CTCs. To confirm that the findings are not model-dependent, we performed blood RNA-seq comparisons between huNBSGW and immunodeficient NSG mice, both injected with the same CTC-derived clonal line, as previously described (1). Transcriptional profiling showed discordance between CTC/CTC clusters according to the presence or absence of human immune cells [Fig. 5B (top)]. Notably, the Reactome-generated list of statistically significant pathways contained B cell–related pathways [Fig. 5B (bottom)]. Further interrogation of CTC:B-cell interactions prompted us to expand our analysis to other tissues, such as the brain (Fig. 5C). Brain tumors from second-generation NBSGW and huNBSGW mice were dissociated and interrogated via RNA-seq. Hierarchical clustering between these samples showed distinct discordance between the samples [Fig. 5C (top)]. Reactome analysis of differential gene expression produced statistically significant pathways, including two B cell–related pathways [Fig. 5C (bottom)]. Few B cells were identified in huNBSGW first-generation brain tumors and first- and second-generation liver tumors (Supplementary Fig. S1A). Altogether, huNBSGW studies showed important clustering between B cells and melanoma CTCs, which was independent of mouse models and tissues.

**Figure 6:**
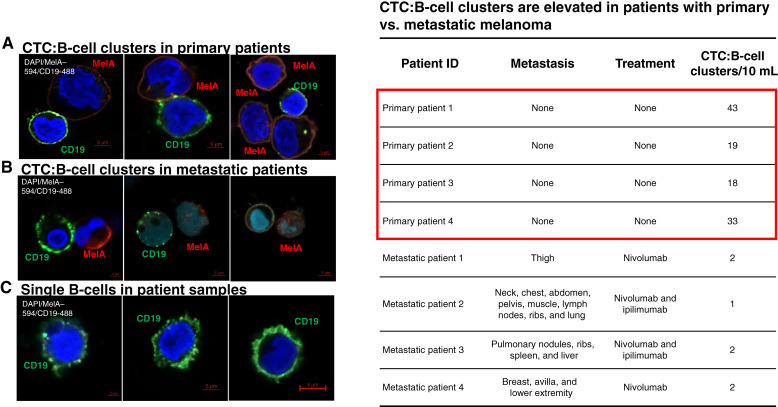
Detection and quantitation of CTC (MelA^+^/DAPI^+^/CD45^−^):B-cell (CD19^+^/DAPI^+^/CD45^+^) clusters in blood of patients with melanoma by Parsortix. Visualization of CTC:B-cell clusters in patients with primary melanoma (**A**), metastatic (**B**) melanoma, and single B cells (**C**) in patient samples (CD19^+^/DAPI^+^/MelA^−^ cells) were performed using high-definition Zeiss 800 confocal microscopy (left). Right, the number of CTC:B-cell clusters/patient sample analyzed. High numbers of heterotypic CTC:B-cell clusters were detected in blood of patients with primary melanoma (red box). Patients’ clinical parameters are shown in Supplementary Table S4. See “Materials and Methods” for experimental details.

To confirm CTC:B-cell clustering in clinical settings, blood from patients with melanoma was immunostained via Parsortix for human CD19-488, human MelA Alexa 594, and DAPI [Fig. 6A–C (left)] staining. CTC:B-cell interactions were visualized and quantified by high-definition confocal microscopy. Blood was provided by patients with primary and metastatic melanoma. Primary patients did not have any clinically diagnosed metastasis and did not receive any treatment or immunotherapy other than surgical removal of the primary tumor in conjunction with nodal staging with sentinel lymph node biopsy (patients’ clinical parameters are shown in Supplementary Table S4). Of relevance, the number of CTC:B-cell clusters in primary patients was 15 to 20-fold higher than that in metastatic patients [Fig. 6A and B (right)]. These observations suggest that CTC:B-cell clusters may be critical in the early stages of CTC dissemination toward metastatic onset.

### B-cell crosstalks with CTC-derived tumor cells by 10x Genomics Xenium

**Figure 7:**
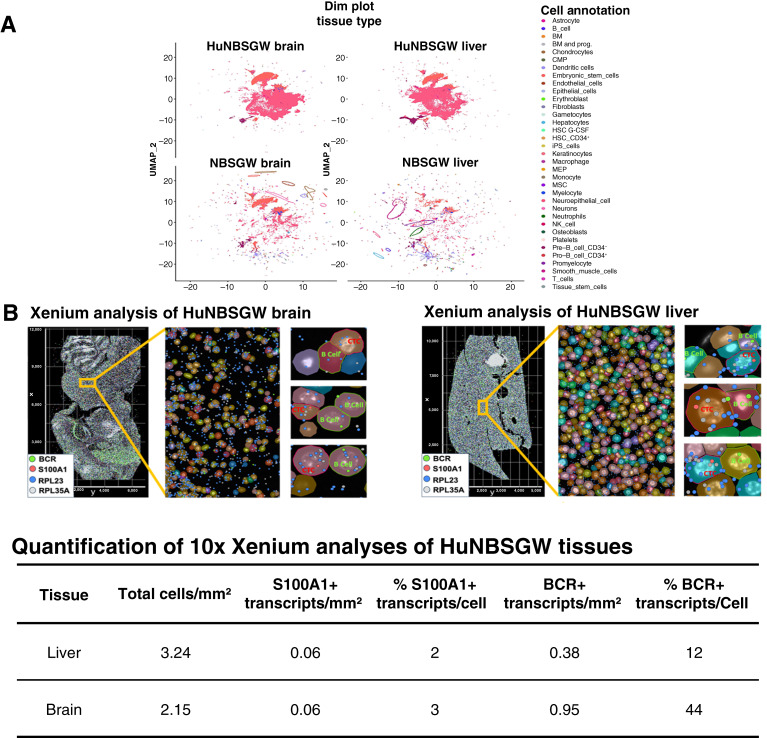
Spatial single-cell gene expression analysis of CTC-derived cell:B-cell interactions employing 10x Genomics Xenium platform (www.10xgenomics.com). HuNBSGW A2.1 mice were engrafted with CTC-derived clonal cells via intracardiac injection (70W-SM3-Luc2; 5.0 × 10^5^ cells/animal). **A,** Dim plot shows 2D scatter plot representing cell clusters in reduced space. Cell annotations are shown on the right. Brain (**B**, top left) and liver (**B**, top right) metastases of second-generation CDXs were isolated, paraffin-embedded, sectioned, and analyzed via the 10x Xenium platform. An image of the whole tissue section of huNBSGW brain (**B**) and huNBSGW liver is presented (left), followed by two close-up images (right). CTC-derived melanoma cells were identified as S100A1^+^ cells (red), whereas B cells were defined as BCR^+^ cells (green). Only melanoma cells showed extensive RPL23^+^ and RPL35A^+^ gene expression as significant members of the RPL/RPS CTC signature (31). Bottom, quantitation of percentage and number of S100^+^ and BCR^+^ cells/mm^2^. See “Materials and Methods” for experimental details. BM, bone marrow; UMAP, Uniform Manifold Approximation and Projection; CMP, common myeloid progenitor; iPS, induced pluripotent stem cell; MEP, Megakaryocyte–erythroid progenitor cell; MSC, mesenchymal stem cell.

**Figure 8:**
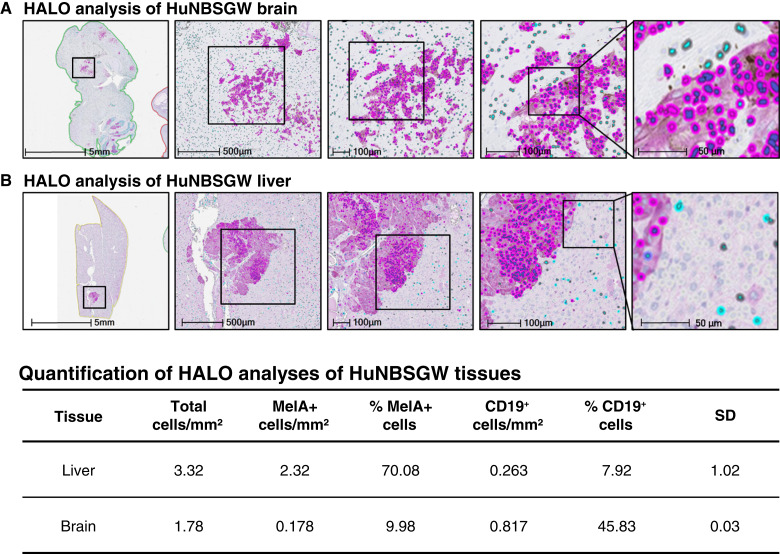
Spatial single-cell protein expression analyses of huNBSGW tumor tissues by HALO platform (Indica Lab). HuNBSGW A2.1 tumor tissues: (**A**) humanized brain and (**B**) humanized liver were cut on Microtome right after the Xenium sections and stained for MelA and CD19 to visualize melanoma and B cells, respectively (top). Positive cells were quantified using HALO software. Bottom panel shows numbers and percentages of MelA^+^ and CD19^+^ cells in the whole section. See “Materials and Methods” for experimental details.

To explore interactions between CTC-derived cells and B cells at the tissues level, we used single-cell spatial transcriptomics (10x Xenium platform). HuNBSGW A2.1 mice were used to match human leukocyte antigen of the CTC-derived clone with the humanized mouse model. Eight huNBSGW A2.1 mice were intracardiacally injected with the CTC-derived clonal cells and imaged using 3D IVIS tomography, confirming metastasis to the brain, liver, and intestine (Supplementary Fig. S5A). Representative brain, liver, and intestine mouse tumors were fixed, paraffin-embedded, sectioned, and used for hematoxylin and eosin staining (Supplementary Fig. S5B), spatial transcriptomics via the single-cell Xenium platform [Fig. 7; Supplementary Fig. S6 (top)], and further protein immunostaining/HALO analysis (Fig. 8; Supplementary Fig. S7). Dim plot analysis of tumor tissues visualized plots of cells in a reduced dimensional space on a 2D scatter plot (Fig. 7A), whereas bar plot analysis determined the percentage of all samples in clusters (Supplementary Fig. S8). Heatmaps of huNBSGW versus NBSGW tissues indicated distinct differences of gene expression in the presence or absence of human immune cells (Supplementary Figs. S9–S11).

The custom-designed 10× Xenium gene panel contained 30 human genes, including pathologically established BCR and S100A1 melanoma markers and RPL23 and RPL35A as the most representative members of the melanoma CTC signature. We have previously reported that RPL23 and RPL35A are two RPL/RPS members of 21 CTC RPL/RPS genes that are downregulated in response to translation and proliferation inhibition (31). These two biomarkers were used to compare RPL/RPS transcript counts in melanoma versus surrounding cells. Spatial transcriptomics confirmed clustering of CTC-derived melanoma cells with B cells in humanized melanoma brain metastasis and liver [Fig. 7B (top left; top right)], liver [Fig. 7 (top right)], and intestine (Supplementary Fig. S6). The number and percentage of S100A1 and BCR transcripts in tumor tissues are shown [Fig. 7 (bottom); Supplementary Fig. S6 (bottom)]. RPL/RPS gene transcript expression was significantly higher in CTC-derived melanoma cells than that in human immune cells (Fig. 7B; Supplementary Fig. S6).

### HALO analysis

To perform an unbiased tissue quantitation, we used the HALO system of the whole-slide imaging analysis of huNBSGW brain tissues with melanoma brain metastasis [Fig. 8A and B (top); Supplementary Fig. S7]. Tissues were stained with MelA and CD19 to visualize human melanoma and B cells, respectively. The whole tissue is shown in the left image of the panels, with close-up views to follow. Quantification of the number of MelA and CD19^+^ B cells was performed using the HALO software [Fig. 8B (bottom)]. Importantly, areas with a high percentage of tumor cells showed intravasation of B cells in those areas. Physical interactions between B cells and tumor cells are visible in the close-up images (right). Collectively, HALO analysis confirmed not only the presence of human B cells in metastatic lesions but also their significant interactions with CTC-derived melanoma cells.

## Discussion

CTCs intravasate into the blood stream from primary and metastatic tumors to establish either primary or secondary metastasis (“metastasis of metastasis”; refs. 14, 51). The development of organ-specific metastasis is not random but rather a complex, continuous, and highly selective process with target organ metastatic specificity unique to each cancer type (14, 52, 53). Melanoma metastasis occurs at multiple sites, including the brain, liver, lung, spleen, pancreas, lymph nodes, and bone (54). In addition, metastasis consists of heterogeneous cell populations which introduce many challenges for selecting a successful treatment. Patients diagnosed with melanoma brain metastasis often present with liver metastasis, and this associates with poor clinical prognosis (55). In this study, by using the newly developed NBSGW mouse model and injections of a melanoma CTC-derived clone in sequential generations of mice (Supplementary Fig. S3 flowchart), we provide for the first time evidence that (i) CTC-driven primary melanoma brain metastasis has target organ specificity to generate extensive secondary metastasis, notably to the liver, supporting the presence of a CTC brain–liver metastasis axis; (ii) the detection and upregulation of CTC:B-cell clusters closely relate to melanoma progression from primary to secondary metastatic disease; and (iii) commonality, association, and upregulation of the RPL/RPS melanoma brain metastasis CTC gene signature were not only significantly maintained but also amplified in CTC secondary liver metastasis of melanoma brain metastasis.

Only original brain tumor (melanoma brain metastasis)–resident cells gave rise to liver metastasis in the second-generation huNBSGW mice as second-generation animals injected with blood from the first-generation blood-injected animals did not develop any metastasis to the liver. For blood-injected animals, only melanoma brain metastasis onset was observed (Fig. 2C). This dichotomy can be ascribed to the distinct properties these cells have before or after intravasation in blood. However, the metastatic pattern for liver metastasis was detected, which recapitulates the clinical characteristics of patients with melanoma, notably the ones diagnosed with melanoma brain metastasis (55). Multiple events must occur for cells to leave the brain and metastasize to the liver, e.g., blood intravasation, survival in the circulation, arrest to the target organ, adhesion, invasion, and micrometastatic onset. All these steps involve complex mechanistic events, which are still unclear for the most part and are the subject of active investigations. Our scope was to delineate the biological events of secondary metastasis by performing “CTC-centric” experiments which centered on the CTC RPL/RPS gene signature of melanoma brain metastasis and the validity of this CTC signature not only for melanoma brain metastasis onset but beyond. We addressed the hypothesis that melanoma brain metastasis CTCs possessing this signature drive extracranial secondary metastasis (“metastasis of metastasis”), notably to the liver, as we have herein demonstrated. Although we cannot prove the direct movement of CTCs from melanoma brain metastasis to the liver, we can state that cells originating from CTC-driven melanoma brain metastasis (first-generation mice) expressing the RPL/RPS gene signature have a predilection to colonize the liver versus other organs, in parallel to augmented expression of the signature. Furthermore, using the sensitive Luc2 reporter, we were able to detect metastases in the brain of these animals 24 hours following injection. Conversely, liver metastases were detected at much later times (10–15 days) in the disease progression. We consider these findings relevant for their clinical implication, e.g., linking melanoma brain metastasis with liver metastasis within the clinical metastatic cascade seen in patients The CTC-driven brain–liver metastasis axis has been postulated in other cancers, e.g., by the injection of Lin-/CTC populations isolated from patients with triple-negative breast cancers diagnosed with brain metastasis in sequential generations of CDXs (immunodeficient NSG mice), without direct confirmation or the presence of functional human immune system cells (56).

The cell populations of the brain tumor microenvironment (TME) are primarily composed of neurons, astrocytes, oligodendrocytes, and microglia, whereas the liver microenvironment is dominated by hepatocytes (parenchymal cells) alongside a large population of nonparenchymal cells, including Kupffer cells (resident macrophages), hepatic sinusoidal endothelial cells, and various other immune cell subsets, making the liver significantly more immune cell–rich compared with the brain. Furthermore, the brain has a highly selective blood–brain barrier restricting the entry of many molecules and immune cells, making the brain microenvironment relatively immune-privileged compared with the liver. Accordingly, the liver TME contains a much broader range of immune cells, including various T-cell subsets, dendritic cells, NK cells, and neutrophils, depending on the physiologic/pathologic state.

It is noteworthy that RNA-seq demonstrated a statistically significant five-fold increase in the RPL/RPS CTC gene signature in melanoma brain metastasis–derived liver metastasis related to a brain–liver metastasis axis (Fig. 3). We surmise that extensive liver metastases had increased RPL/RPS gene expression to alter ribosome structure, composition, and function, e.g., “onco-ribosomes” promoting augmented protein translation, oncogenic signaling pathways, and/or other cellular properties required for the multiple metastatic steps (57). Furthermore, increased ribosomal synthesis is also linked to metabolic plasticity which is pivotal for tumor cell survival and the tolerance of oxidative/nitrosative stress (50, 55). To this end, we have previously reported that dual *in vivo* targeting of both CTC translation and proliferation is critical to rewire the metabolic plasticity of CTCs (31). The discrimination of onco-ribosome structural composition, protein–protein interactions, and their function (active vs. dormant types) is currently underway and involves the use of cryogenic electron microscopy (ref. 58).

Another important finding of this study is that melanoma brain metastasis–derived liver metastasis was detected only in humanized mice and associated with CTC:B-cell clustering. We used a nonirradiated huNBSGW mouse model that accurately recapitulates the genetic heterogeneity of human immune cell populations and recruits immune cells to tumor sites (38). Our data show that humanized immune cells clearly had an impact on melanoma progression in this in vivo model which more closely reflects the complexity of the blood microenvironment/TME found in patients. By performing unbiased analyses of peripheral blood from patients with primary (blood collected adjacent to the primary lesion) and metastatic melanoma using the FDA-approved CTC Parsortix platform, we detected high numbers of CTC:B-cell clusters explicitly in the former with a 15/20-fold decrease in the latter [Fig. 6 (right)]. In addition, although parallel cohorts of first- and second-generation NBSGW mice were treated exactly in the same conditions as huNBSGW mice, not only a lower percentage of NBSGW mice developed liver metastasis versus humanized NBSGW mice (20% vs. 40%) but also at a significantly decreased size, e.g., macro- versus micro-metastasis (latter defined as at least 0.2 mm but no larger than 0.2 mm or 200 cells; Fig. 2B). With the engraftment of luc-tagged CTC-derived clonal cells in huNBSGW mice, human tumor-associated macrophages and tumor-infiltrating lymphocytes were detected in brain and liver tumors (Supplementary Fig. S1). Future studies will determine the mechanistic underpinnings of the role of B cells in supporting the progression of melanoma brain metastasis CTCs to promote liver metastasis.

The presence of immune cells in the huNBSGW mouse allowed us to explore CTC clusters in these mice. CTC clusters comprise only 2% to 5% of all CTCs; however, the degree of the disease severity, either clinically or preclinically, is directly proportional to their number (24, 25, 27). Of these, heterotypic CTC clusters include normal immune cells, such as PMN-MDSCs, neutrophils, CD3 cells, CD4 cells, and platelets (25–27, 35). To the best of our knowledge, this study reports for the first time evidence of heterotypic CTC:B-cell clusters associated with clinical or experimental melanoma metastasis (Figs. 5 and 6). B cells can contribute to innate and adaptive immune responses, underscoring the relevance of understanding the function of various subsets of B cells in metastasis (36, 59). For example, a recent study has demonstrated that B-cell infiltration in the TME is linked to poor clinical outcomes and cancer immunotherapy resistance (60). Prometastatic roles of B cells have been reported in other cancer types, such as ovarian and bladder cancers (61–64). Tumor-infiltrating B cells induce the secretion of the proangiogenic marker lymphotoxin, which activates NF-κB signaling and promotes tumor progression (65–67). Additionally, B cells enable tumor development through the production of tumor growth factors, such as TGFβ, IL-10, and IL-35 (67). We extended these findings by detecting and analyzing interactions between melanoma cells/CTCs with B cells either in blood (CTC clusters) or CTC-derived primary/secondary metastasis by single-cell gene or protein expression (10× Genomics Xenium and HALO spatial biology systems, respectively; Figs. 7 and 8). The cross-talk between the immune microenvironment and cancer cells is highly complex, but our findings suggest that the interrogation of CTC:B-cell cluster functionalities may serve as a prognostic factor in the systemic evaluation of cancer progression and determination of an appropriate treatment approach.

This study has some limitations. First, we analyzed a limited number of patients with primary and metastatic melanoma. Therefore, we cannot conclude that the observed heterotypic CTC:B-cell clusters will be detected in the blood of all patients with melanoma. Similarly, the use of a single CTC-derived clone may not faithfully recapitulate the extensive CTCs/tumor heterogeneity detected in patients with melanoma. Second, we did not directly associate the presence of increased RPL/RPS gene expression with the presence of CTC:B-cell clusters. Mechanistic studies to modulate this signature as a result of CTC:B-cell clustering are warranted. Third, we did not characterize specific B-cell subclusters, B-cell signaling, and/or B-cell regulatory functionalities which may modulate tumor progression. The identification of specific B-cell subclusters can be relevant to assess disease severity and to prescribe more suitable treatment for an individual patient. Lastly, although we performed experiments in which a primary tumor was established with second-generation cells by subcutaneous injection in huNBSGW and NBSGW mice, mice had to be euthanized because of the size of primary subcutaneous (SQ) tumors (humane end point of experiment) before they developed any metastases. Very few single CTCs were detected in blood of either huNBSGW or NBSGW mice at the time of necropsy, preventing further analyses. Additional investigations are warranted to address these limitations. Regardless, this study reports for the first time evidence of a brain–liver metastasis axis which associates with the presence of B cells acting as traveling partners of melanoma CTCs and foster additional investigations of their roles in primary and secondary metastases. There are consistently fewer B-cell frequencies in distant sites versus the primary tumor across metastatic sites (36, 68). However, the precise mechanisms that underpin B-cell clustering with CTCs and/or the reduced B-cell surveillance in the metastatic sites are critical but yet to be fully evaluated. A potential interpretation of our findings is that CTC-derived cell:B-cell clusters intravasate from melanoma brain metastasis once generated, which marks the high percentage of B cells in huNBSGW brain relative to that in livers [Fig. 7 (bottom)], which is extremely relevant in secondary metastatic disease. Their definition could provide an important conduit for translation in the clinic and the development of effective therapeutic agents to improve melanoma patient care.

## Supplementary Material

Figure S1 TAMs and TILs in Livers and Brains

Figure S2 IVIS quantification of the1st generation CDXs

Figure S3 Flowchart of experimental strategy

Figure S4 IVIS MBM quantification of the second generation CDX

Figure S5 IVIS determination to metastasis to distant organs

Figure S6 Xenium analyses of HuNBSGW intestine

Figure S7 HALO analyses of HuNBSGW intestine

Figure S8 Bar plots analyses of 10x Xenium clusters

Figure S9 Heat maps of humanized vs non-humanized brain tissues

Figure S10 Heat maps of humanized vs non-humanized liver tissues

Figure S11 Heat maps of humanized vs non-humanized intestine tissues

Table S1 Table S1

Table S3 Table S3

Table S4 Table S4

Table S2 Table S2
