# Supplementary figures and images for: A Melanoma Brain Metastasis CTC Signature and CTC:B-cell Clusters Associate with Secondary Liver Metastasis: A Melanoma Brain–Liver Metastasis Axis
Source: Cancer Res Commun. 2025 Feb 12;5(2):295–308. doi: 10.1158/2767-9764.CRC-24-0498 (PMC11816052; doi:10.1158/2767-9764.CRC-24-0498)

## Slide 1
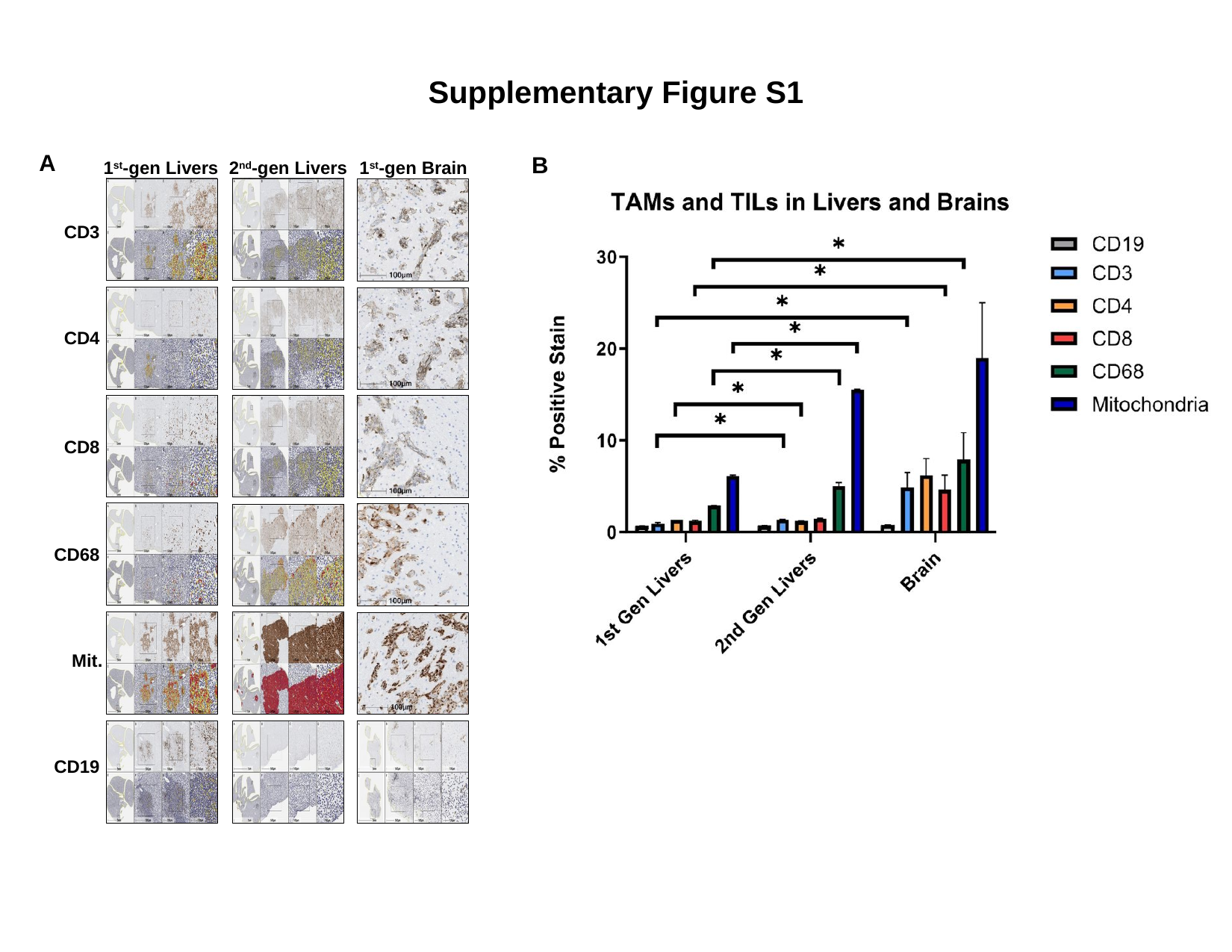

Supplementary Figure S1
A
B
1st-gen Brain
1st-gen Livers
2nd-gen Livers
CD3
CD4
CD8
CD68
Mit.
CD19

Supplement: Figure S1 — TAMs and TILs in Livers and Brains [file crc-24-0498_figure_s1_suppsf1.pptx]
