# Supplementary material for: A Melanoma Brain Metastasis CTC Signature and CTC:B-cell Clusters Associate with Secondary Liver Metastasis: A Melanoma Brain–Liver Metastasis Axis
Source: Cancer Res Commun. 2025 Feb 12;5(2):295–308. doi: 10.1158/2767-9764.CRC-24-0498 (PMC11816052; doi:10.1158/2767-9764.CRC-24-0498)
Supplement: Figure S2 — IVIS quantification of the1st generation CDXs [file crc-24-0498_figure_s2_suppsf2.pptx]

## Slide 1
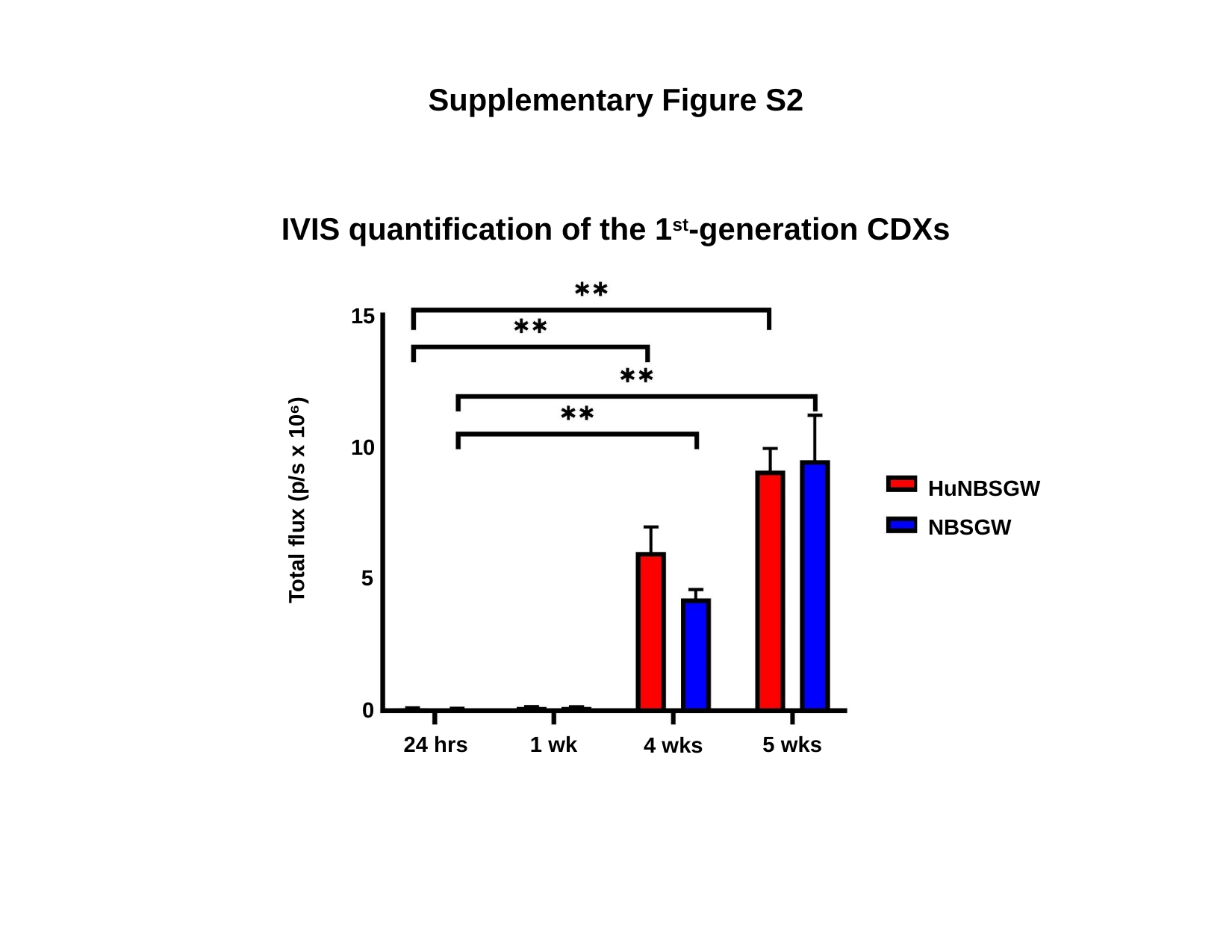

Supplementary Figure S2
IVIS quantification of the 1st-generation CDXs
5 wks
1 wk
24 hrs
4 wks
15
10
HuNBSGW
NBSGW
Total flux (p/s x 10⁶)
5
0
