# Supplementary material for: A Melanoma Brain Metastasis CTC Signature and CTC:B-cell Clusters Associate with Secondary Liver Metastasis: A Melanoma Brain–Liver Metastasis Axis
Source: Cancer Res Commun. 2025 Feb 12;5(2):295–308. doi: 10.1158/2767-9764.CRC-24-0498 (PMC11816052; doi:10.1158/2767-9764.CRC-24-0498)
Supplement: Figure S3 — Flowchart of experimental strategy [file crc-24-0498_figure_s3_suppsf3.pptx]

## Slide 1
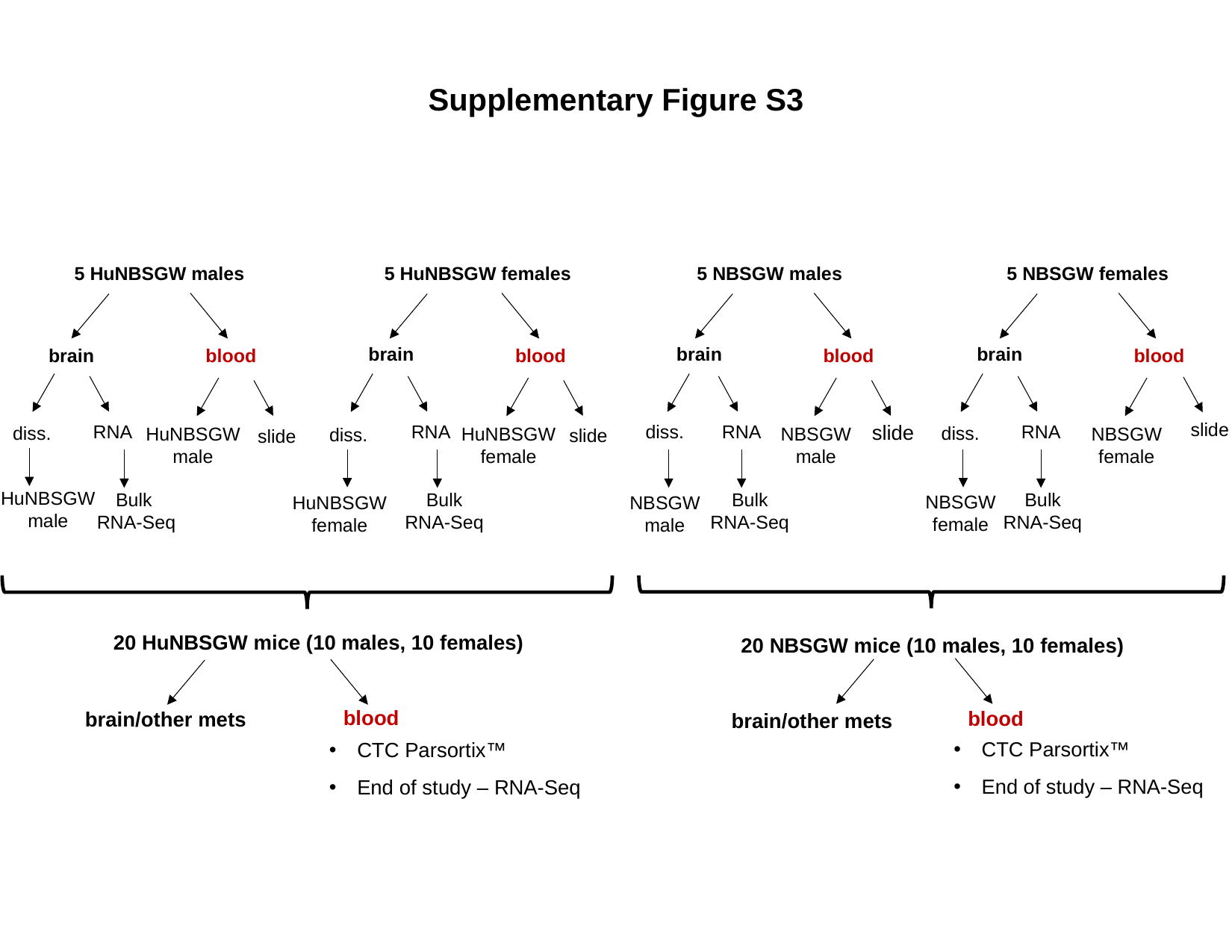

Supplementary Figure S3
5 NBSGW females
brain
blood
RNA
diss.
NBSGW female
Bulk
RNA-Seq
NBSGW female
5 NBSGW males
brain
blood
RNA
diss.
NBSGW male
Bulk
RNA-Seq
NBSGW male
5 HuNBSGW males
blood
brain
RNA
diss.
HuNBSGW male
slide
HuNBSGW male
Bulk
RNA-Seq
5 HuNBSGW females
brain
blood
RNA
HuNBSGW female
diss.
Bulk
RNA-Seq
HuNBSGW female
slide
slide
slide
20 HuNBSGW mice (10 males, 10 females)
20 NBSGW mice (10 males, 10 females)
blood
blood
brain/other mets
brain/other mets
CTC Parsortix™
End of study – RNA-Seq
CTC Parsortix™
End of study – RNA-Seq
