# Supplementary material for: A Melanoma Brain Metastasis CTC Signature and CTC:B-cell Clusters Associate with Secondary Liver Metastasis: A Melanoma Brain–Liver Metastasis Axis
Source: Cancer Res Commun. 2025 Feb 12;5(2):295–308. doi: 10.1158/2767-9764.CRC-24-0498 (PMC11816052; doi:10.1158/2767-9764.CRC-24-0498)
Supplement: Figure S4 — IVIS MBM quantification of the second generation CDX [file crc-24-0498_figure_s4_suppsf4.pptx]

## Slide 1
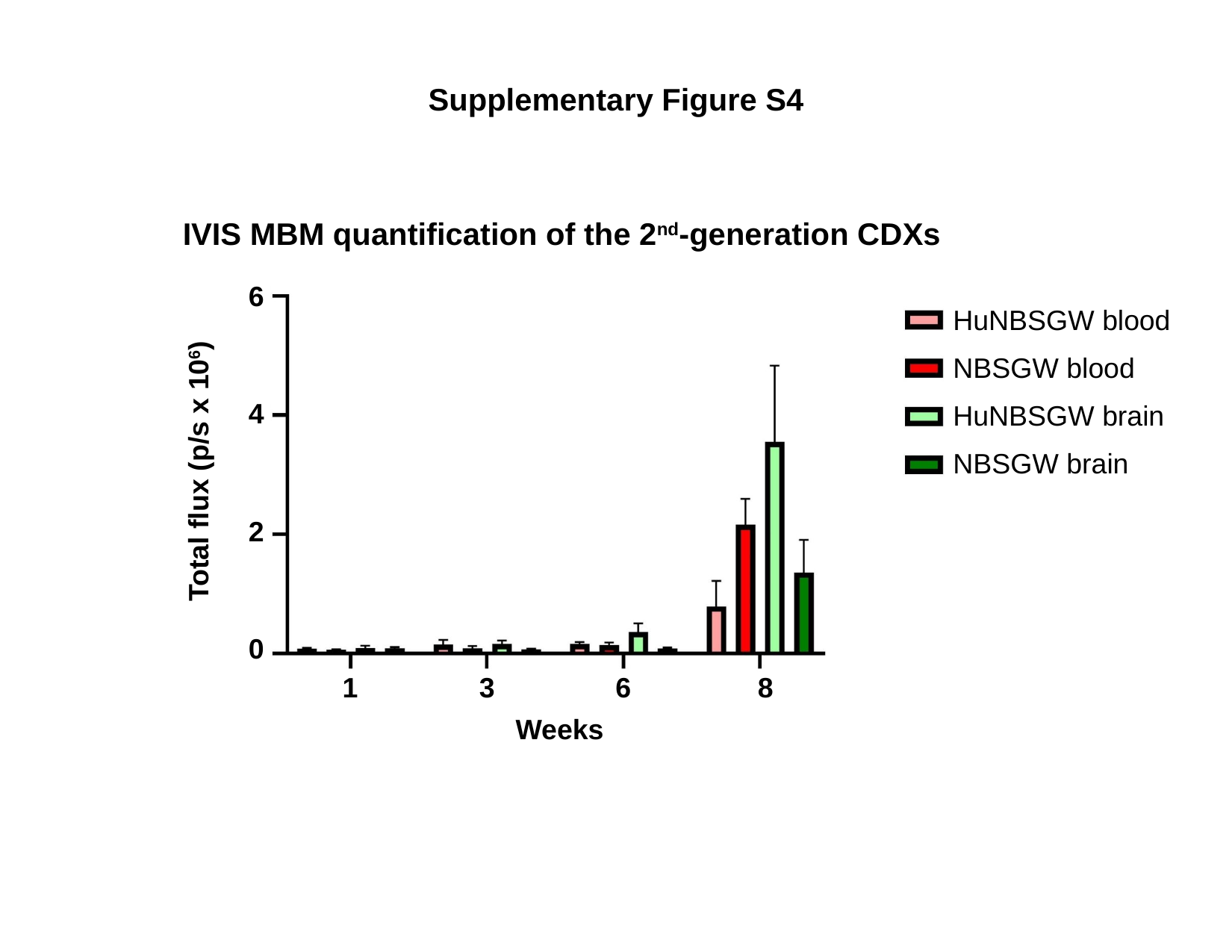

Supplementary Figure S4
IVIS MBM quantification of the 2nd-generation CDXs
6
4
2
0
HuNBSGW blood
NBSGW blood
HuNBSGW brain
NBSGW brain
Total flux (p/s x 106)
1
3
6
8
Weeks
