# Supplementary material for: A Melanoma Brain Metastasis CTC Signature and CTC:B-cell Clusters Associate with Secondary Liver Metastasis: A Melanoma Brain–Liver Metastasis Axis
Source: Cancer Res Commun. 2025 Feb 12;5(2):295–308. doi: 10.1158/2767-9764.CRC-24-0498 (PMC11816052; doi:10.1158/2767-9764.CRC-24-0498)
Supplement: Figure S5 — IVIS determination to metastasis to distant organs [file crc-24-0498_figure_s5_suppsf5.pptx]

## Slide 1
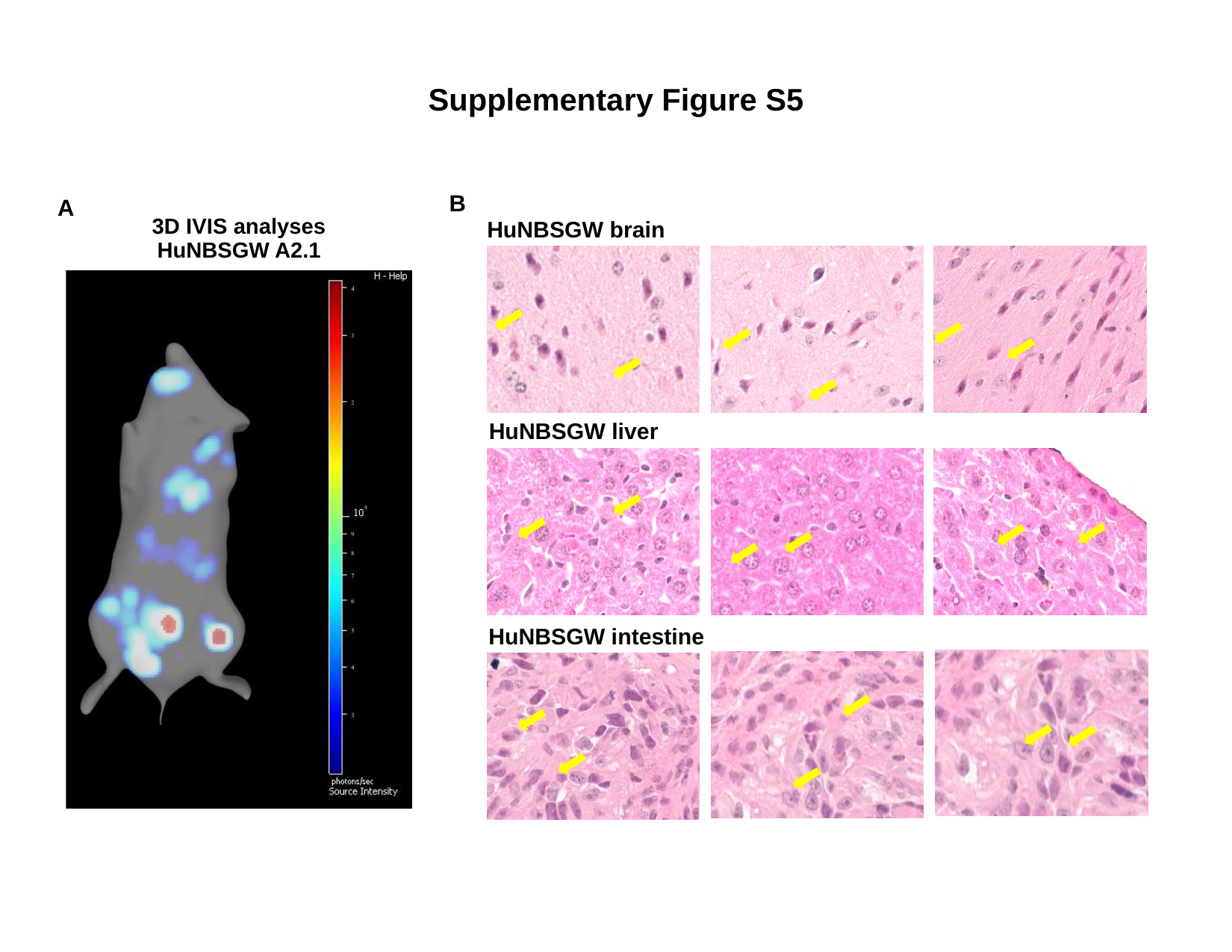

Supplementary Figure S5
B
A
3D IVIS analyses
HuNBSGW A2.1
HuNBSGW brain
HuNBSGW liver
HuNBSGW intestine
