# Supplementary material for: A Melanoma Brain Metastasis CTC Signature and CTC:B-cell Clusters Associate with Secondary Liver Metastasis: A Melanoma Brain–Liver Metastasis Axis
Source: Cancer Res Commun. 2025 Feb 12;5(2):295–308. doi: 10.1158/2767-9764.CRC-24-0498 (PMC11816052; doi:10.1158/2767-9764.CRC-24-0498)
Supplement: Figure S6 — Xenium analyses of HuNBSGW intestine [file crc-24-0498_figure_s6_suppsf6.pptx]

## Slide 1
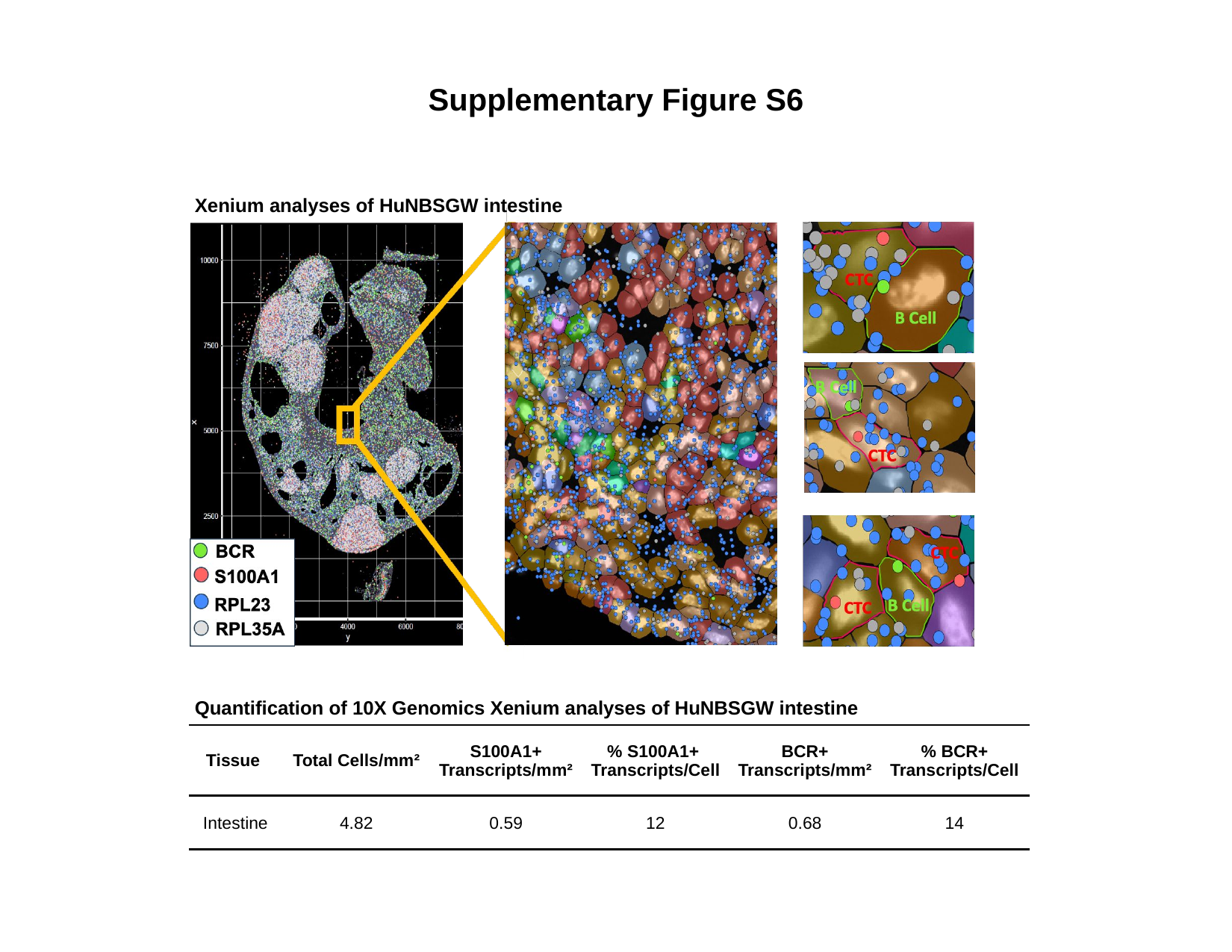

Supplementary Figure S6
Xenium analyses of HuNBSGW intestine
Quantification of 10X Genomics Xenium analyses of HuNBSGW intestine
| Tissue | Total Cells/mm² | S100A1+ Transcripts/mm² | % S100A1+ Transcripts/Cell | BCR+ Transcripts/mm² | % BCR+ Transcripts/Cell |
| --- | --- | --- | --- | --- | --- |
| Intestine | 4.82 | 0.59 | 12 | 0.68 | 14 |
