# Supplementary material for: A Melanoma Brain Metastasis CTC Signature and CTC:B-cell Clusters Associate with Secondary Liver Metastasis: A Melanoma Brain–Liver Metastasis Axis
Source: Cancer Res Commun. 2025 Feb 12;5(2):295–308. doi: 10.1158/2767-9764.CRC-24-0498 (PMC11816052; doi:10.1158/2767-9764.CRC-24-0498)
Supplement: Figure S7 — HALO analyses of HuNBSGW intestine [file crc-24-0498_figure_s7_suppsf7.pptx]

## Slide 1
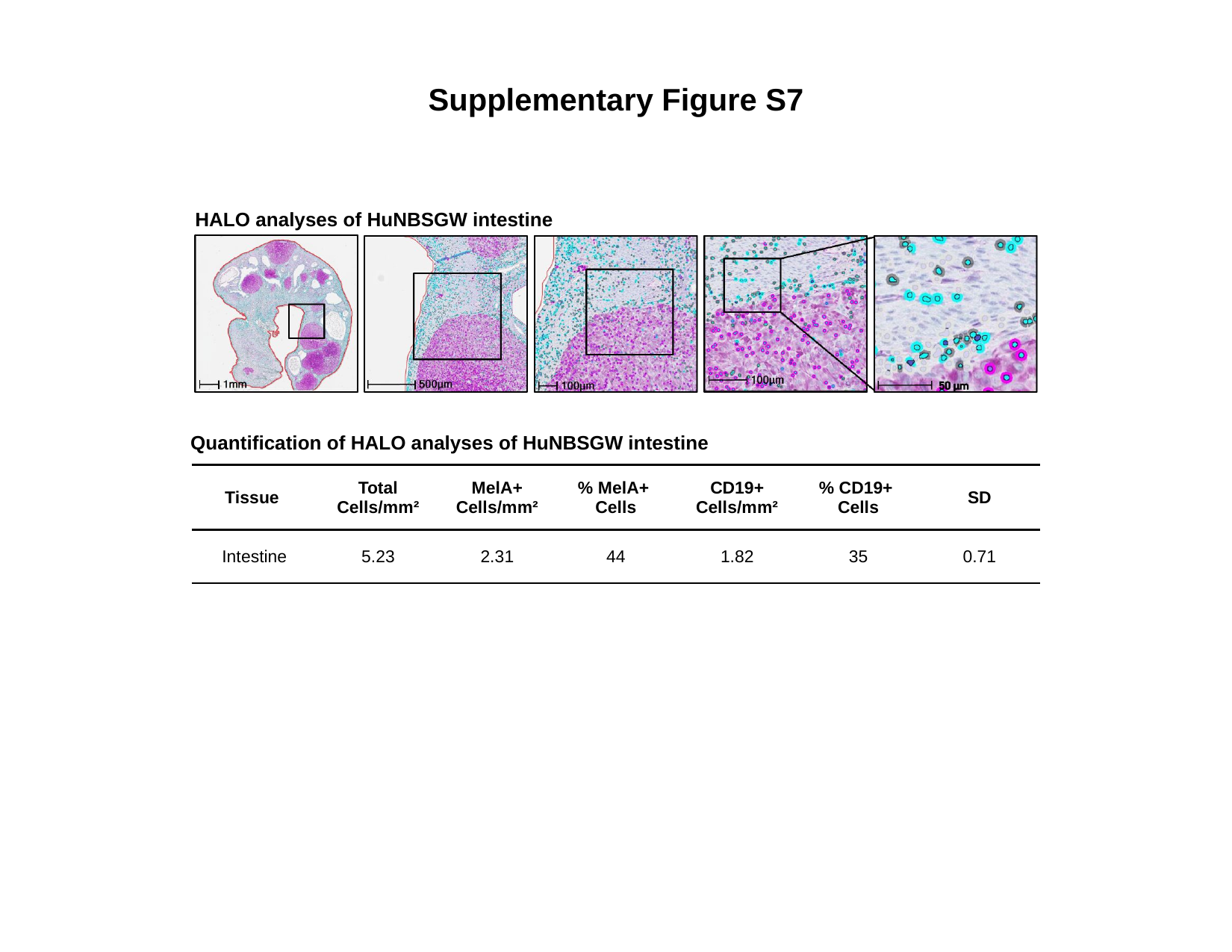

Supplementary Figure S7
HALO analyses of HuNBSGW intestine
Quantification of HALO analyses of HuNBSGW intestine
| Tissue | Total Cells/mm² | MelA+ Cells/mm² | % MelA+ Cells | CD19+ Cells/mm² | % CD19+ Cells | SD |
| --- | --- | --- | --- | --- | --- | --- |
| Intestine | 5.23 | 2.31 | 44 | 1.82 | 35 | 0.71 |
