# Supplementary material for: A Melanoma Brain Metastasis CTC Signature and CTC:B-cell Clusters Associate with Secondary Liver Metastasis: A Melanoma Brain–Liver Metastasis Axis
Source: Cancer Res Commun. 2025 Feb 12;5(2):295–308. doi: 10.1158/2767-9764.CRC-24-0498 (PMC11816052; doi:10.1158/2767-9764.CRC-24-0498)
Supplement: Figure S8 — Bar plots analyses of 10x Xenium clusters [file crc-24-0498_figure_s8_suppsf8.pptx]

## Slide 1
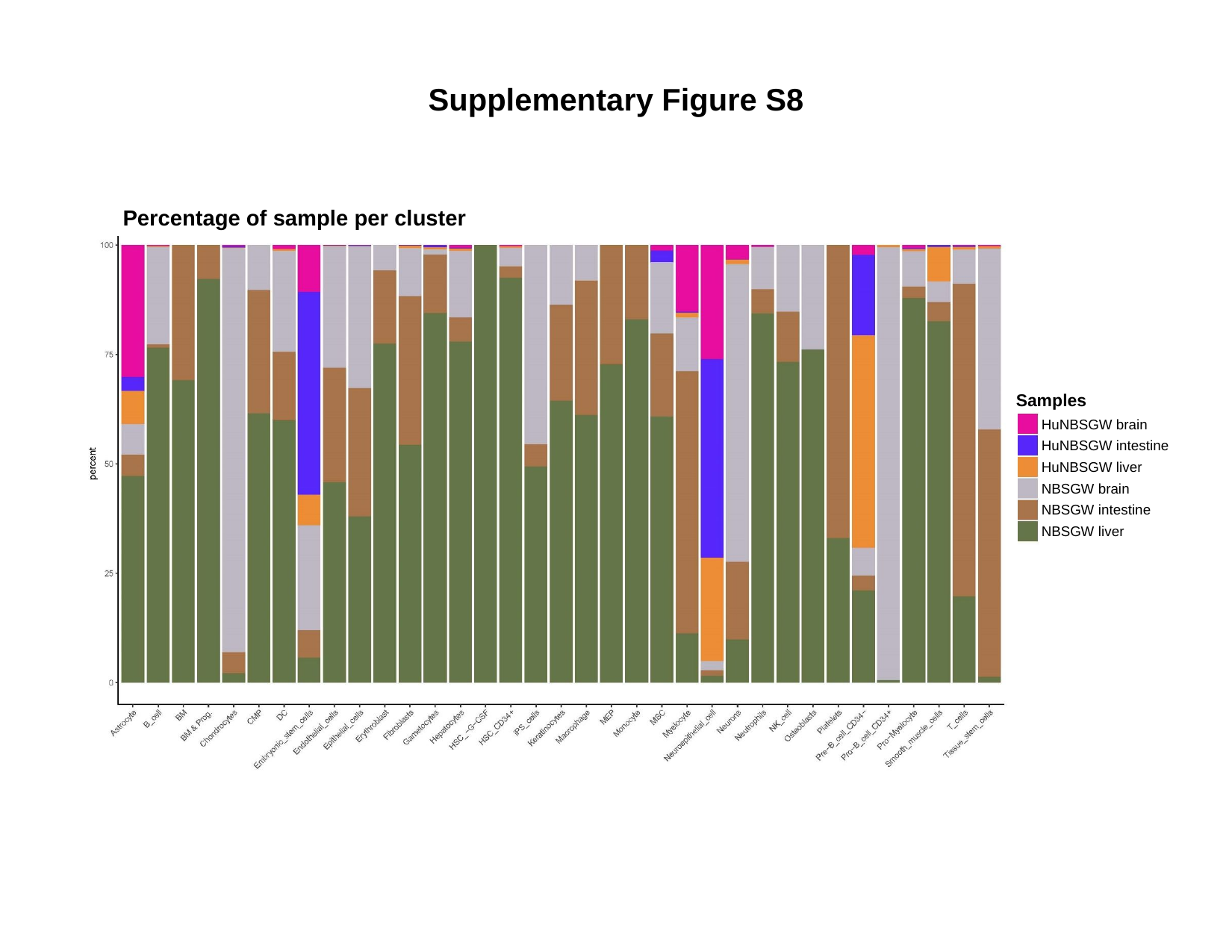

Supplementary Figure S8
Percentage of sample per cluster
Samples
HuNBSGW brain
HuNBSGW intestine
HuNBSGW liver
NBSGW brain
NBSGW intestine
NBSGW liver
