# Supplementary material for: A Melanoma Brain Metastasis CTC Signature and CTC:B-cell Clusters Associate with Secondary Liver Metastasis: A Melanoma Brain–Liver Metastasis Axis
Source: Cancer Res Commun. 2025 Feb 12;5(2):295–308. doi: 10.1158/2767-9764.CRC-24-0498 (PMC11816052; doi:10.1158/2767-9764.CRC-24-0498)
Supplement: Table S1 [file crc-24-0498_table_s1_suppst1.pptx]

## Slide 1
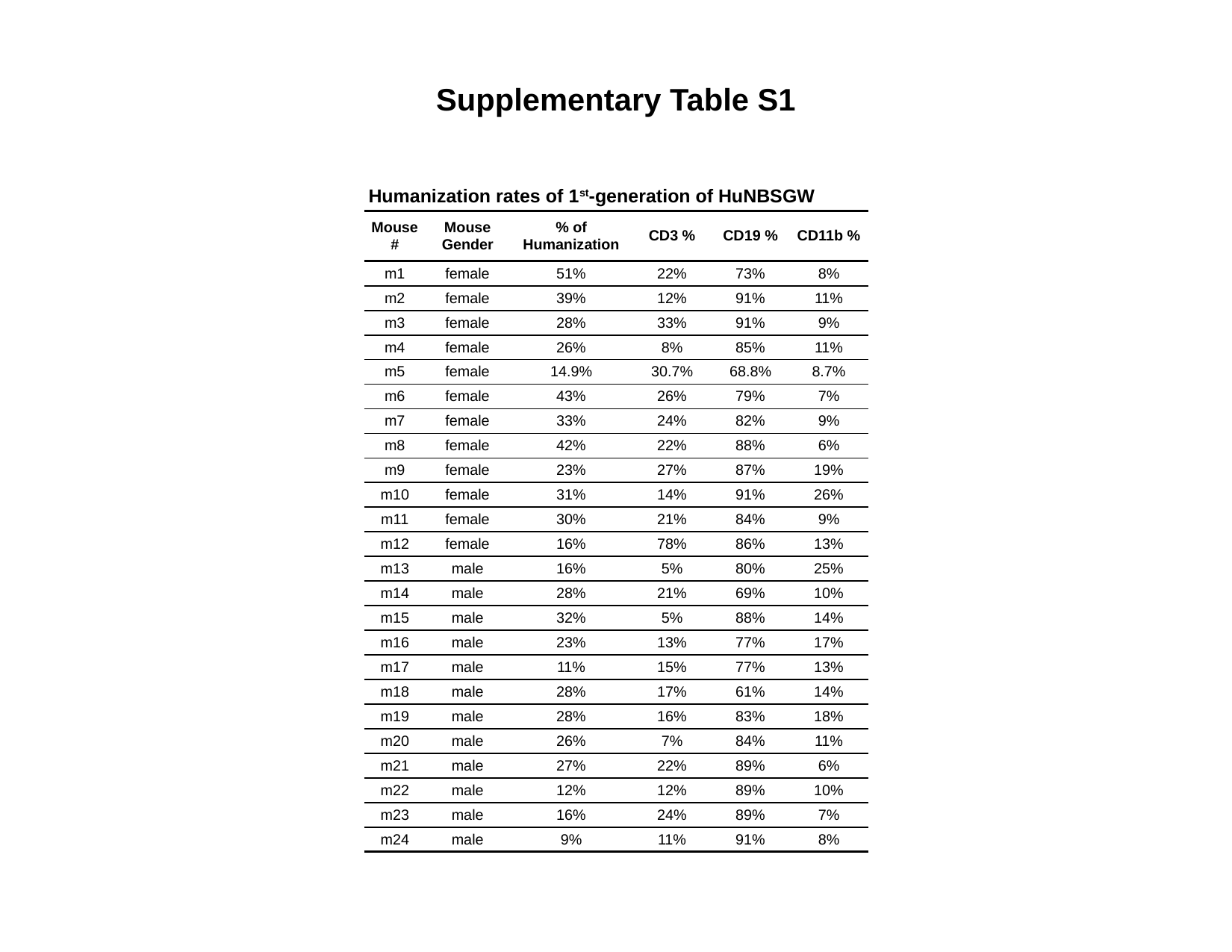

Supplementary Table S1
# Humanization rates of 1st-generation of HuNBSGW
| Mouse # | Mouse Gender | % of Humanization | CD3 % | CD19 % | CD11b % |
| --- | --- | --- | --- | --- | --- |
| m1 | female | 51% | 22% | 73% | 8% |
| m2 | female | 39% | 12% | 91% | 11% |
| m3 | female | 28% | 33% | 91% | 9% |
| m4 | female | 26% | 8% | 85% | 11% |
| m5 | female | 14.9% | 30.7% | 68.8% | 8.7% |
| m6 | female | 43% | 26% | 79% | 7% |
| m7 | female | 33% | 24% | 82% | 9% |
| m8 | female | 42% | 22% | 88% | 6% |
| m9 | female | 23% | 27% | 87% | 19% |
| m10 | female | 31% | 14% | 91% | 26% |
| m11 | female | 30% | 21% | 84% | 9% |
| m12 | female | 16% | 78% | 86% | 13% |
| m13 | male | 16% | 5% | 80% | 25% |
| m14 | male | 28% | 21% | 69% | 10% |
| m15 | male | 32% | 5% | 88% | 14% |
| m16 | male | 23% | 13% | 77% | 17% |
| m17 | male | 11% | 15% | 77% | 13% |
| m18 | male | 28% | 17% | 61% | 14% |
| m19 | male | 28% | 16% | 83% | 18% |
| m20 | male | 26% | 7% | 84% | 11% |
| m21 | male | 27% | 22% | 89% | 6% |
| m22 | male | 12% | 12% | 89% | 10% |
| m23 | male | 16% | 24% | 89% | 7% |
| m24 | male | 9% | 11% | 91% | 8% |
