# Supplementary material for: A Melanoma Brain Metastasis CTC Signature and CTC:B-cell Clusters Associate with Secondary Liver Metastasis: A Melanoma Brain–Liver Metastasis Axis
Source: Cancer Res Commun. 2025 Feb 12;5(2):295–308. doi: 10.1158/2767-9764.CRC-24-0498 (PMC11816052; doi:10.1158/2767-9764.CRC-24-0498)
Supplement: Table S3 [file crc-24-0498_table_s3_suppst3.pptx]

## Slide 1
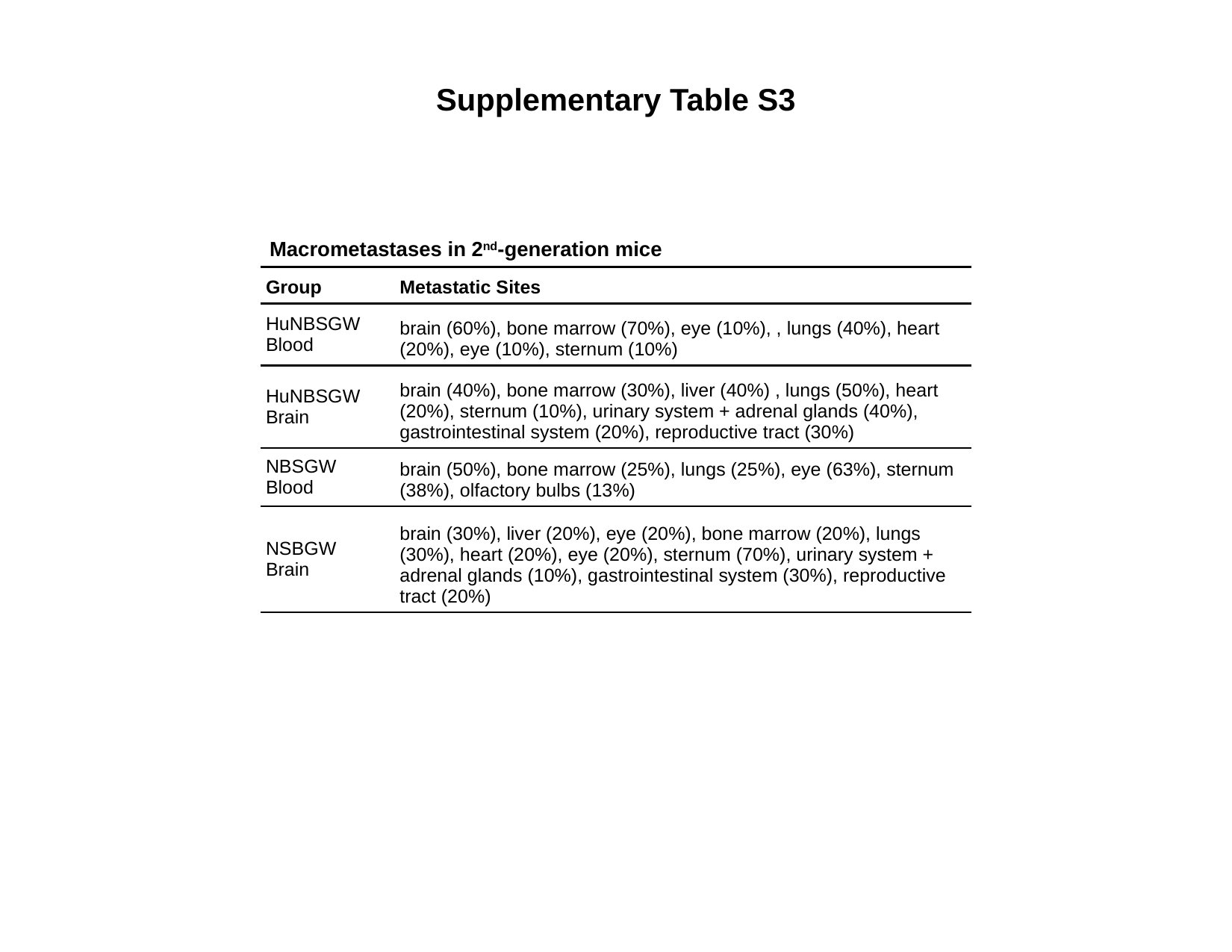

Supplementary Table S3
Macrometastases in 2nd-generation mice
| Group | Metastatic Sites |
| --- | --- |
| HuNBSGW Blood | brain (60%), bone marrow (70%), eye (10%), , lungs (40%), heart (20%), eye (10%), sternum (10%) |
| HuNBSGW Brain | brain (40%), bone marrow (30%), liver (40%) , lungs (50%), heart (20%), sternum (10%), urinary system + adrenal glands (40%), gastrointestinal system (20%), reproductive tract (30%) |
| NBSGW Blood | brain (50%), bone marrow (25%), lungs (25%), eye (63%), sternum (38%), olfactory bulbs (13%) |
| NSBGW Brain | brain (30%), liver (20%), eye (20%), bone marrow (20%), lungs (30%), heart (20%), eye (20%), sternum (70%), urinary system + adrenal glands (10%), gastrointestinal system (30%), reproductive tract (20%) |
