# Supplementary material for: A Melanoma Brain Metastasis CTC Signature and CTC:B-cell Clusters Associate with Secondary Liver Metastasis: A Melanoma Brain–Liver Metastasis Axis
Source: Cancer Res Commun. 2025 Feb 12;5(2):295–308. doi: 10.1158/2767-9764.CRC-24-0498 (PMC11816052; doi:10.1158/2767-9764.CRC-24-0498)
Supplement: Table S4 [file crc-24-0498_table_s4_suppst4.pptx]

## Slide 1
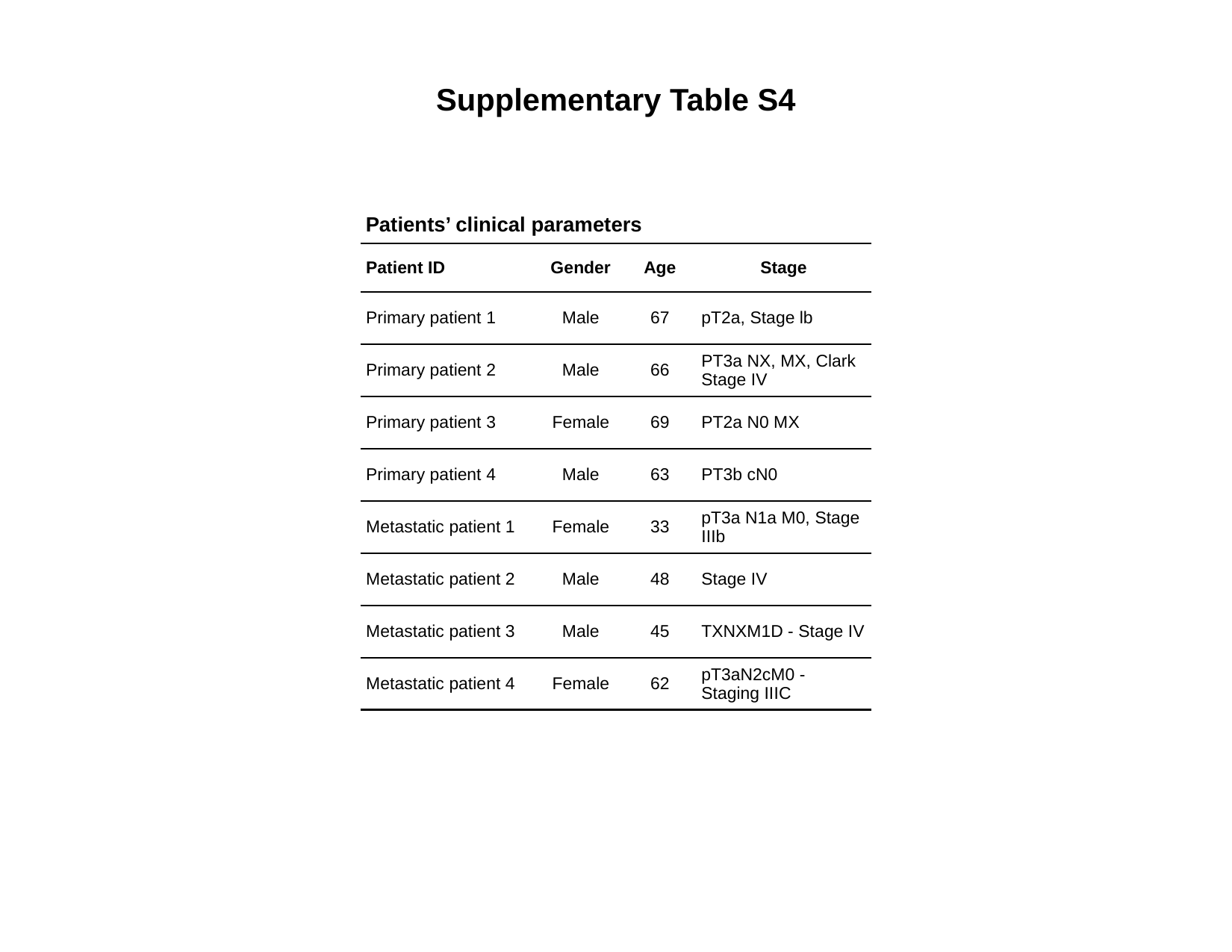

Supplementary Table S4
Patients’ clinical parameters
| Patient ID | Gender | Age | Stage |
| --- | --- | --- | --- |
| Primary patient 1 | Male | 67 | pT2a, Stage lb |
| Primary patient 2 | Male | 66 | PT3a NX, MX, Clark Stage IV |
| Primary patient 3 | Female | 69 | PT2a N0 MX |
| Primary patient 4 | Male | 63 | PT3b cN0 |
| Metastatic patient 1 | Female | 33 | pT3a N1a M0, Stage IIIb |
| Metastatic patient 2 | Male | 48 | Stage IV |
| Metastatic patient 3 | Male | 45 | TXNXM1D - Stage IV |
| Metastatic patient 4 | Female | 62 | pT3aN2cM0 - Staging IIIC |
