# Supplementary material for: A Melanoma Brain Metastasis CTC Signature and CTC:B-cell Clusters Associate with Secondary Liver Metastasis: A Melanoma Brain–Liver Metastasis Axis
Source: Cancer Res Commun. 2025 Feb 12;5(2):295–308. doi: 10.1158/2767-9764.CRC-24-0498 (PMC11816052; doi:10.1158/2767-9764.CRC-24-0498)
Supplement: Table S2 [file crc-24-0498_table_s2_suppst2.pptx]

## Slide 1
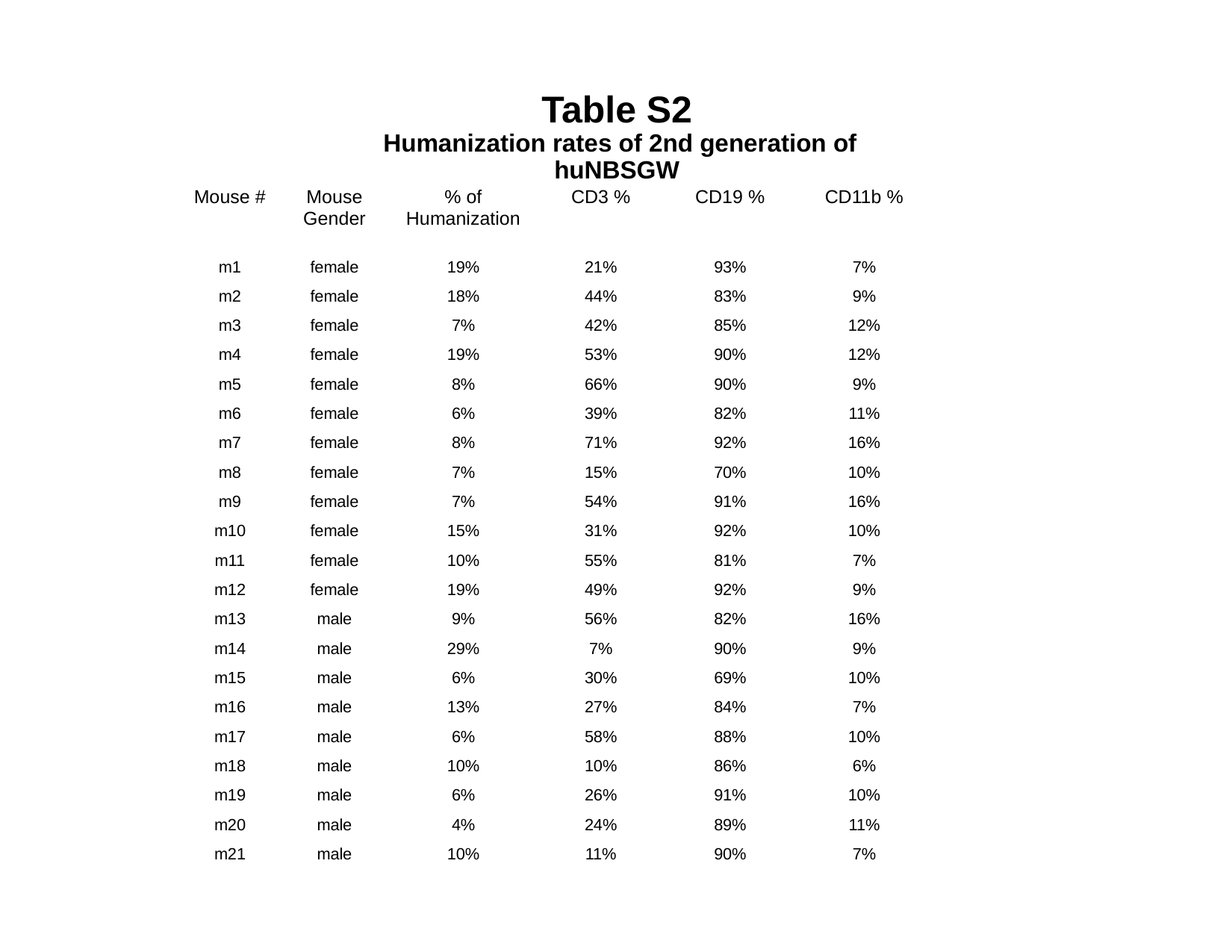

Table S2
 Humanization rates of 2nd generation of huNBSGW
| Mouse # | Mouse Gender | % of Humanization | CD3 % | CD19 % | CD11b % |
| --- | --- | --- | --- | --- | --- |
| m1 | female | 19% | 21% | 93% | 7% |
| m2 | female | 18% | 44% | 83% | 9% |
| m3 | female | 7% | 42% | 85% | 12% |
| m4 | female | 19% | 53% | 90% | 12% |
| m5 | female | 8% | 66% | 90% | 9% |
| m6 | female | 6% | 39% | 82% | 11% |
| m7 | female | 8% | 71% | 92% | 16% |
| m8 | female | 7% | 15% | 70% | 10% |
| m9 | female | 7% | 54% | 91% | 16% |
| m10 | female | 15% | 31% | 92% | 10% |
| m11 | female | 10% | 55% | 81% | 7% |
| m12 | female | 19% | 49% | 92% | 9% |
| m13 | male | 9% | 56% | 82% | 16% |
| m14 | male | 29% | 7% | 90% | 9% |
| m15 | male | 6% | 30% | 69% | 10% |
| m16 | male | 13% | 27% | 84% | 7% |
| m17 | male | 6% | 58% | 88% | 10% |
| m18 | male | 10% | 10% | 86% | 6% |
| m19 | male | 6% | 26% | 91% | 10% |
| m20 | male | 4% | 24% | 89% | 11% |
| m21 | male | 10% | 11% | 90% | 7% |
